# Combined CDK2 and CDK4/6 Inhibition Overcomes Palbociclib Resistance in Breast Cancer by Enhancing Senescence

**DOI:** 10.3390/cancers12123566

**Published:** 2020-11-29

**Authors:** Kamal Pandey, Nahee Park, Kyung-Soon Park, Jin Hur, Yong Bin Cho, Minsil Kang, Hee-Jung An, Sewha Kim, Sohyun Hwang, Yong Wha Moon

**Affiliations:** 1 Hematology and Oncology, Department of Internal Medicine, CHA Bundang Medical Center, CHA University, Seongnam 13488, Korea; pkamal@chauniv.ac.kr (K.P.); skgml0413@naver.com (N.P.); hurjinz@naver.com (J.H.); ybyoungbin@naver.com (Y.B.C.); rbtnr1@naver.com (M.K.); 2 Department of Biomedical Science, CHA University, Seongnam 13488, Korea; kspark@cha.ac.kr (K.-S.P.); blissfulwin@cha.ac.kr (S.H.); 3 Department of Pathology, CHA Bundang Medical Center, CHA University, Seongnam 13488, Korea; hjahn@cha.ac.kr (H.-J.A.); sewhakim@chamc.co.kr (S.K.)

**Keywords:** CDK4/6, CDK2, hormone receptor-positive breast cancer, drug resistance, C-MYC

## Abstract

**Simple Summary:**

Cyclin-dependent kinase 4 and 6 (CDK4/6) inhibitors are widely used to treat metastatic hormone receptor-positive/human epidermal growth factor receptor 2-negative breast cancer. Despite the effectiveness of CDK4/6 inhibitors, acquired resistance occurs in almost all cases. Strategies to address this issue have not been developed yet. We investigated mechanisms of resistance to CDK4/6 inhibitor in breast cancer and potential therapeutic strategies. We found that cyclin E-CDK2 mediated phosphorylation of C-MYC is responsible for resistance to CDK4/6 inhibitor by suppressing C-MYC induced senescence. On the contrary, the synergistic anti-proliferative effect of the combined inhibition of CDK2 and CDK4/6 overcomes acquired resistance to CDK4/6 inhibitors by enhancing senescence. Our findings could pave the way for the development CDK2-specific kinase inhibitor for the treatment of breast cancers that are resistant to CDK4/6 inhibitor.

**Abstract:**

Breast cancer represents the number one global cancer burden in women and the hormone receptor (HR)-positive subtype comprises approximately 70% of breast cancers. Unfortunately, acquired resistance ultimately occurs in almost all cases, even though cyclin-dependent kinase 4 and 6 (CDK4/6) inhibitors are a highly effective therapy for HR-positive/human epidermal growth factor receptor 2-negative subtype. Here, we investigated mechanisms of resistance to CDK4/6 inhibitor and potential therapeutic strategies using our palbociclib-resistant preclinical model. We observed that cyclin E was significantly overexpressed in palbociclib-resistant cells, and similar association was also confirmed in pleural effusion samples collected from HR-positive breast cancer patients. After confirmation of cyclin E-CDK2 interaction by co-immunoprecipitation, we demonstrated CDK2 inhibition combined with palbociclib synergistically suppressed proliferation of palbociclib-resistant cells and growth of palbociclib-resistant xenograft in mice. We also proved that enhancing C-MYC-mediated senescence is a novel mechanism behind the synergism created by targeting both CDK2 and CDK4/6. Furthermore, the clinical relevance of cyclin E as a therapeutic target was supported by significant association between CCNE1 overexpression and poor prognosis based on large-scale public gene expression data sets in HR-positive breast cancer patients. Therefore, we propose cyclin E-CDK2 signaling as a promising therapeutic target for overcoming cyclin E-associated resistance to CDK4/6 inhibitor.

## 1. Introduction

Breast cancer remains a major health concern and exhibits the highest cancer-related mortality among women worldwide [1]. Based on the status of the hormone receptor (HR) and the human epidermal growth factor receptor 2 (HER2), HR-positive breast cancer is the major subtype comprising about 70% of all breast cancers [2]. Although endocrine therapy is considered to be the main therapy, acquired resistance has notably evolved, which is an important issue in the treatment of this type of cancer that needs to be addressed [3,4].

Dysregulation of the cyclin D-cyclin-dependent kinase (CDK)4/6-retinoblastoma protein (RB) pathway causes uncontrolled proliferation of cells and has been implicated in the pathogenesis of HR-positive breast cancer [5], i.e., RB acts as a tumor suppressor by interacting with the E2F transcription factor and blocks its transcriptional activity. However, phosphorylation of RB by the cyclin D-CDK4/6 complex impairs its ability to interact with E2F, which allows E2F to be transcriptionally active and promote cancer progression [6]. Therefore, inhibition of the CDK4/6 pathway has emerged as a promising strategy to treat HR-positive/HER2-negative breast cancer. In HR-positive/HER2-negative metastatic breast cancer, CDK4/6 inhibitors combined with an aromatase inhibitor are used as first-line therapy [7], and CDK4/6 inhibitors combined with fulvestrant are used as second-line therapy when the aromatase inhibitor treatment fails to prolong progression-free survival [8,9,10].

Although CDK4/6 inhibitors show a promising outcome, acquired resistance develops in almost all cases after 24–28 months when first-line therapy is used [7,11,12] and after a shorter period when second-line therapy is used [8,9,10]. Multiple mechanisms of resistance to CDK4/6 inhibitors have been previously described [13]. Cell cycle-related resistance mechanisms include the loss of RB [14], p16 amplification [15] and CDK6 or CDK4 amplification [16], and bypass pathway-related resistant mechanisms include activation of FGFR [17] or PI3K/AKT/mTOR [14]. Apart from CDK4/6 inhibitors, various other CDK inhibitors including CDK1, CDK7 and CDK9 are being studied and are under different stages of development [18]. In particular, a recent preclinical study has suggested that targeting CDK7 could overcome CDK4/6 inhibitor resistance in HR-positive breast cancer [19]. However, there are no established methods to overcome resistance. 

The overexpression of cyclin E has been proposed as one of the potential mechanisms of resistance to CDK4/6 inhibitor [14,20,21]. A recent study analyzing pre-treatment tumor samples derived from the PALOMA-3 trial identified the overexpression of cyclin E as a mechanism by which breast cancer escapes the effects of palbociclib [22]. Cyclin E forms a complex with CDK2 to regulate G1-S cell cycle progression, [23] and activation of the cyclin E-CDK2 pathway could compensate for CDK4/6 inhibition via a bypass mechanism. Taken together, cyclin E-CDK2 theoretically could be a resistance mechanism and therapeutic target in CDK4/6 inhibitor-resistant cases. 

In this study, we investigated mechanisms of acquired resistance to CDK4/6 inhibitor, focusing on the cyclin E-CDK2 pathway. Furthermore, we demonstrated that the combined inhibition of CDK2 and CDK4/6 could be a promising approach for treating palbociclib-resistant breast cancer.

## 2. Results

### 2.1. Generation and Confirmation of Palbociclib-Resistant Cell Lines

**Figure 1:**
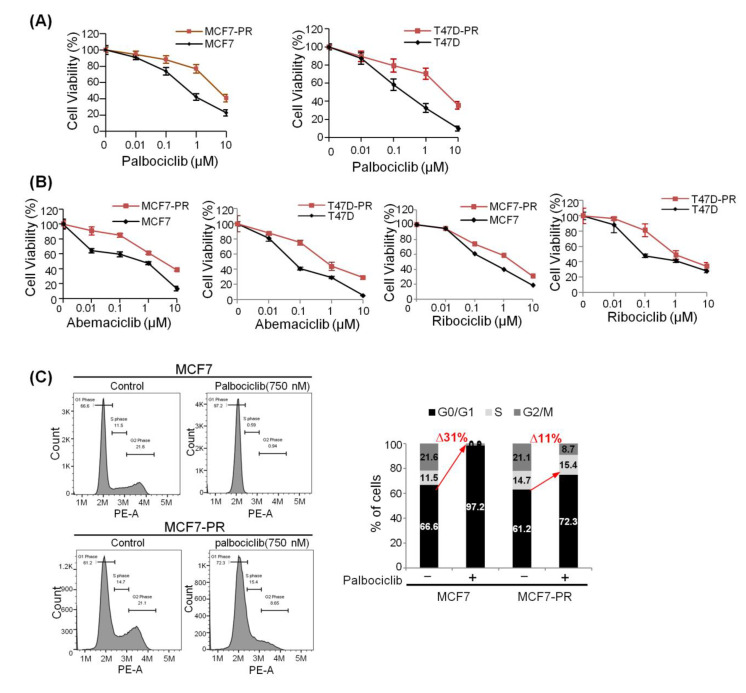
Derivation and confirmation of resistance to CDK4/6 inhibitor using HR-positive cells. (**A**,**B**) The viability of MCF7, MCF7-PR, T47D, and T47D-PR cells after treatment with palbociclib at different concentrations for 6 days was assessed by MTT. (**B**) Palbociclib-resistant cells were cross resistant with other CDK4/6 inhibitors (abemaciclib and ribociclib). The cell viability assay was performed, as mentioned earlier. (**C**) MCF7 and MCF7-PR cells were treated with palbociclib (750 nM) for 48 h, and a cell cycle analysis was performed. The histogram represents the distribution of cells in the G0/G1, S, and G2/M phase, and the bar graph indicates the percentage of cells in the G0/G1, S, and G2/M phase of the cell cycle.

We generated palbociclib-resistant HR-positive breast cancer cell lines as described in the Methods section (Figure S1A). A significant increase was observed in the IC_50_ of palbociclib in palbociclib-resistant cells (7.15 µM in MCF7-PR and 3.37 µM in T47D-PR) relative to their corresponding parental cells (0.75 µM in MCF7 and 0.26 µM in T47D) (Figure 1A). The palbociclib-resistant cells also showed cross resistance with other CDK4/6 inhibitors, such as abemaciclib and ribociclib (Figure 1B). Recent evidence suggests that epithelial to mesenchymal transition (EMT) is responsible for the gain of resistance to CDK4/6 inhibitor [13]. We observed the significant overexpression of ZEB1, Vimentin, and N-cadherin in MCF7-PR cells, whereas the expression of E-cadherin was significantly lower in MCF7-PR cells (Figure S1B,C). Moreover, the morphology of MCF7-PR cells was more similar to mesenchymal cells compared with their corresponding parental cells, which exhibited a typical epithelial morphology (Figure S1D). EMT phenomenon provided us the intriguing evidence that it could be a critical mediator of drug resistance. Therefore, we plan to do another project separately with EMT inhibitor using this model. We performed cell cycle analysis to compare the effectiveness of palbociclib in causing G1 arrest. Palbociclib could not block the resistant cells at the G1 phase, whereas parental cells were arrested at G1 by palbociclib. Moreover, a significant increase in the proportion of MCF7-PR cells in the S phase suggests that the resistant cells may have bypassed the CDK4/6 inhibition (Figure 1C and Figure S2).

### 2.2. RB Loss and Cyclin E Overexpression Are Observed in CDK4/6 Inhibitor-Resistant Cells

**Figure 2:**
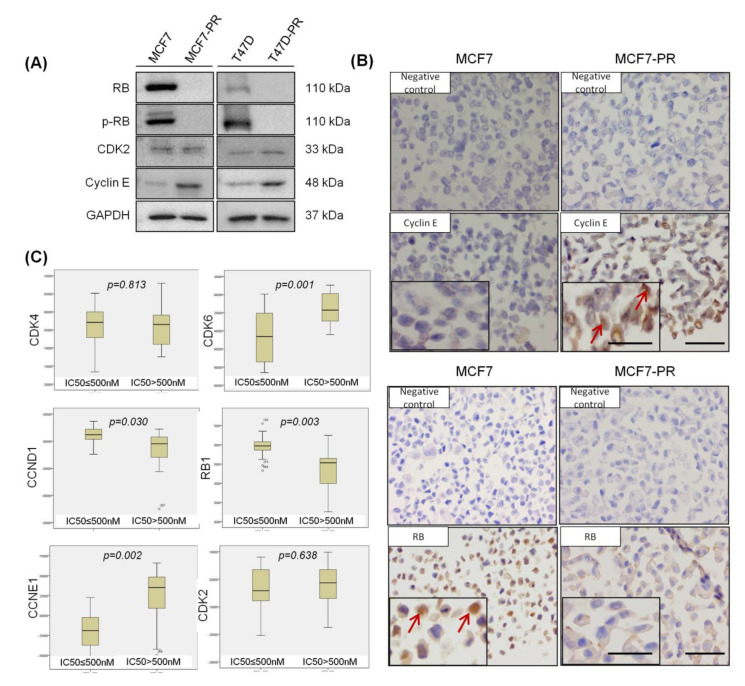
Alteration of cell cycle-related genes and protein expression in palbociclib-resistant cells. (**A**) Western blot analysis of indicated antibodies was performed. A list of antibodies is given in Table S1. (**B**) Immunohistochemistry also confirmed that cyclin E was overexpressed, and RB was lost in palbociclib-resistant cell blocks compared with sensitive cell blocks. Cell blocks were generated using MCF7 and MCF7-PR cells. Red arrows in the figure denote the positively expressed cells of the indicated proteins (magnification 200×, scale bars = 100 µm; 400×, scale bars = 50 µm) (**C**) Correlation of CCLE cell cycle genes and palbociclib sensitivity, which was defined as IC_50_ ≤ 500 nM, in breast cancer cell lines. *p*-values were calculated by student’s *t*-tests. Full length blots **(A)** are presented in Figure S5.

Various preclinical studies reported that the loss of RB and overexpression of cyclin E are predictive biomarkers for palbociclib resistance [6,13]. When we compared the expression of cell cycle-related proteins between palbociclib-resistant cells and their sensitive counterparts, we also observed the significant overexpression of cyclin E and loss of RB in the palbociclib-resistant cells (Figure 2A,B). Furthermore, in the analysis of the relationship between palbociclib activity and cell cycle-related gene expression in 38 breast cancer cell lines out of Cancer Cell Line Encyclopedia (CCLE) data [24]. We found that the cells with low palbociclib activity (IC_50_ > 500 nM) had higher cyclin E expression (*p* = 0.002) (Figure 2C). These results suggest that cyclin E plays a specific role in mediating resistance to CDK4/6 inhibitor.

### 2.3. CDK2 Inhibitor Synergizes with Palbociclib to Inhibit Cell Proliferation 

**Figure 3:**
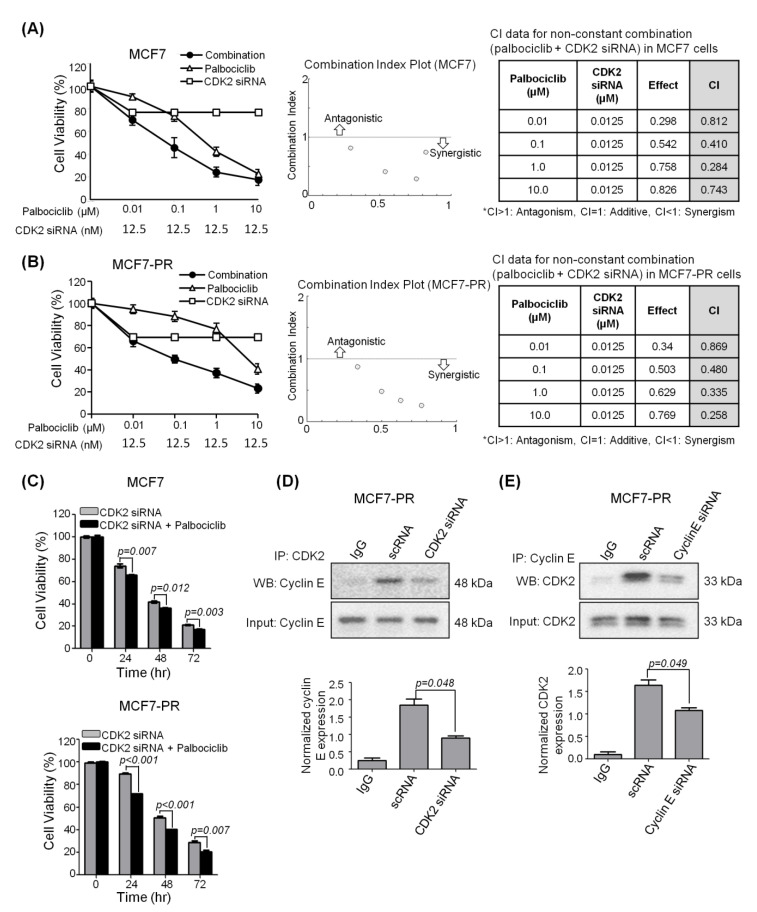
CDK2 inhibitor synergizes with palbociclib to inhibit cell proliferation. (**A**,**B**) MTT assay showed that 12.5 nM of CDK2 siRNA augmented the antiproliferative effect in combination with various concentrations of palbociclib in (**A**) MCF7 and (**B**) MCF7-PR cells. (**C**) The combined inhibition of CDK2 (12.5 nM) and palbociclib (750 nM) at various treatment time points. *p*-values were calculated by student’s *t*-test at each time point. Data are presented as the mean± S.E.M. (**D**) The complex formation of cyclin E and CDK2 was measured by the co-immunoprecipitation assay. The complex in MCF7-PR cells was impaired by CDK2 siRNA treatment (12.5 nM for 72 h). *p*-values were calculated by a paired *t*-test. Data are presented as the mean ± S.E.M. of triplicate experiments. (**E**) The complex in MCF7-PR cells was impaired by cyclin E siRNA treatment (10 nM for 72 h). *p*-values were calculated by a paired *t*-test. Data are presented as the mean ± S.E.M. of triplicate experiments. Full length blots (**D**,**E**) are presented in Figure S5.

Based on the fact that cyclin E overexpression is a biomarker of resistance to CDK4/6 inhibitor [6,25], we examined whether inhibition of cyclin E-CDK2 signaling synergizes with CDK4/6 inhibition. We treated MCF7 and MCF7-PR cells with CDK2 siRNA (12.5 nM) and different concentrations of palbociclib. The combined inhibition of CDK2 and CDK4/6 synergistically reduced the cell proliferation of MCF7 (CI < 1) and MCF7-PR cells (CI < 1) compared with the inhibition of CDK2 or CDK4/6 alone (Figure 3A–C and Figure S3). We next assessed the direct interaction between CDK2 and cyclin E using a co-immunoprecipitation assay. Firstly, to investigate if the inhibition of CDK2 breaks the complex, we treated MCF7-PR cells with CDK2 siRNA and a scrambled siRNA control. We found that following CDK2 knockdown, the protein expression of cyclin E was reduced (Figure 3D) (*p* = 0.048). Furthermore, the interaction of cyclin E and CDK2 was confirmed by reverse CO-IP (Figure 3E). Taken together, these results suggest that inhibition of CDK2 can prevent the formation of the cyclin E-CDK2 complex, thereby inhibiting the proliferation of cyclin E-overexpressed cancer cells.

### 2.4. Inhibition of CDK2 Increases Senescence by Inhibiting Phospho-C-MYC, which Is Responsible for Acquired Resistance to Palbociclib

**Figure 4:**
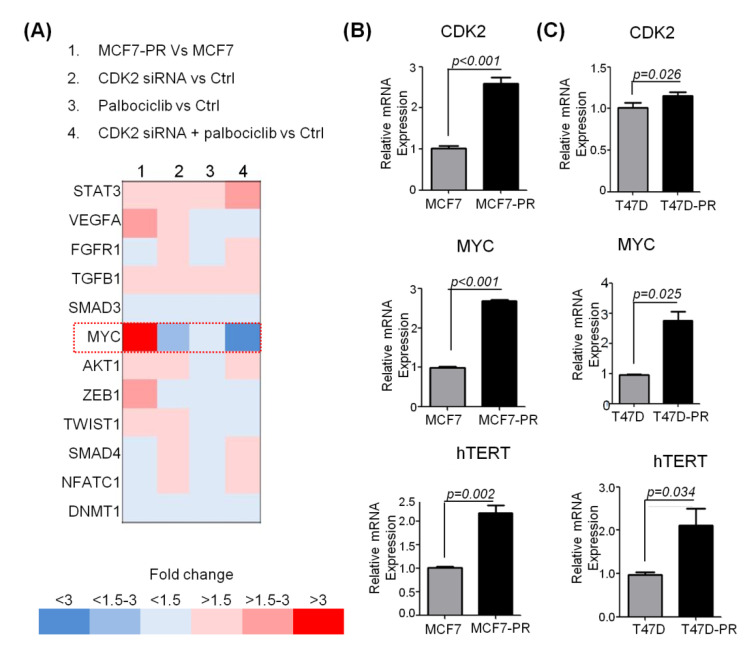
Up-regulation of the C-MYC gene in MCF7-PR cells. (**A**) Microarray analysis was performed on MCF7 and MCF7-PR cells after treatment with CDK2 siRNA (12.5 nM), palbociclib (750 nM) and their combination for 48 h. We selected the genes that could have been involved in resistance to CDK4/6 inhibitor by referring to our previously published article [13]. Gene expression analysis revealed that C-MYC was up-regulated in MCF7-PR cells compared with MCF7 cells, down-regulated by CDK2 siRNA, and further down-regulated by the combination treatment. (**B**) CDK2, C-MYC, and hTERT were up-regulated in MCF7-PR and (**C**) T47D-PR cells compared with their sensitive counterparts, which was determined by qRT-PCR. *p*-values were calculated by student’s *t*-test. Data are presented as the mean ± S.E.M. of triplicate experiments. A list of primers is given in Table S2**.**

To identify the mechanisms underlying the synergism of the combined inhibition of CDK2 and CDK4/6, we compared gene expression profiles after treating both sensitive and resistant cells with palbociclib, CDK2 siRNA, and a combination of the two. We observed that C-MYC was significantly up-regulated in resistant cells (3.1-fold increased; *p* < 0.001). Interestingly, overexpressed C-MYC was significantly suppressed by inhibiting CDK2 (2.2-fold decreased; *p* < 0.001) and further suppressed by inhibiting both CDK2 and CDK4/6 (3.0-fold decreased; *p* < 0.001) (Figure 4A). Overexpression of C-MYC in the palbociclib-resistant cells was validated with qRT-PCR (MCF7-PR vs. MCF7; 2.7-fold increased; *p* < 0.001 and T47D-PR vs. T47D; 2.8-fold increased; *p* = 0.025) (Figure 4B).

C-MYC is a broad-range oncogenic transcription factor that promotes the progression of cancer cells [26]. The C-MYC target genes, hTERT, which is a senescence-blocking gene [27], increased in palbociclib-resistant cells compared with palbociclib-sensitive cells (MCF7-PR vs. MCF7; 2.2-fold increased; *p* = 0.002 and T47D-PR vs. T47D; 2.1-fold increased; *p* = 0.034) (Figure 4B). Furthermore, CDK4/6 and CDK2 was known to phosphorylate C-MYC at ser62, which stabilizes C-MYC to transcribe hTERT [28,29,30]. Subsequently, hTERT suppresses C-MYC-induced senescence [27], resulting in cancer progression. 

**Figure 5:**
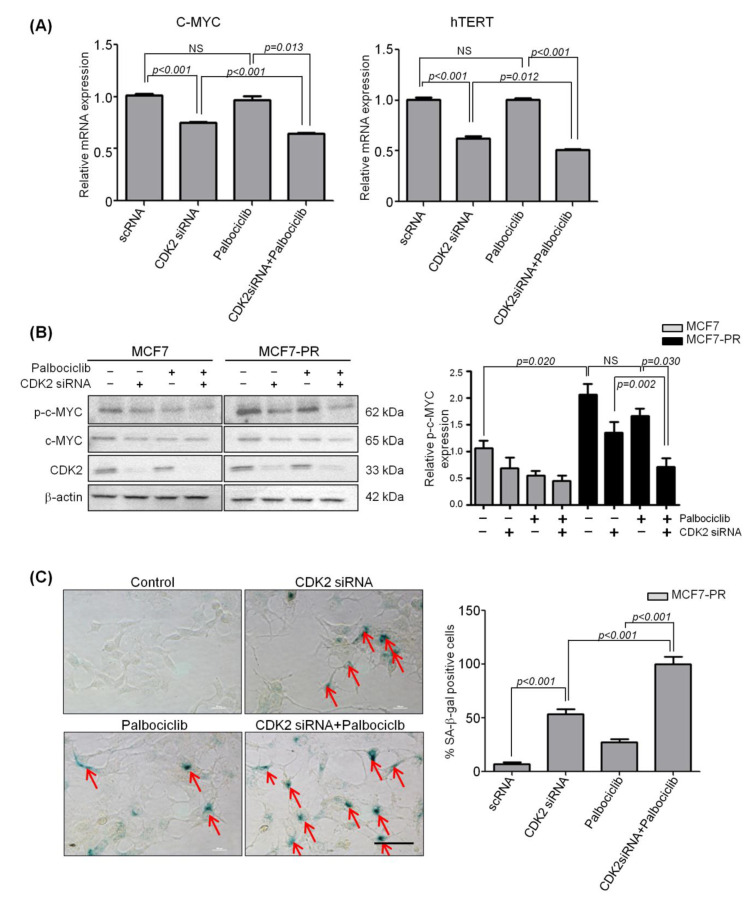
CDK2 phosphorylation of C-MYC promotes cells to escape senescence. (**A**) The relative mRNA expression determined by qRT-PCR. C-MYC and hTERT in MCF7-PR cells were down-regulated by CDK2 siRNA (12.5 nM) or palbociclib (750 nM) and further down-regulated when treated with a combination of the two. *p*-values were calculated by student’s *t*-test. NS indicates not significant. (**B**) Phospho-C-MYC (ser62) decreased in MCF7 and MCF7-PR cells by CDK2 siRNA alone and in combination with palbociclib. *p*-values were calculated by student’s *t*-test. Data are presented as the mean ± S.E.M. of triplicate experiments. NS indicates not significant. (**C**) SA-β-galactosidase staining using MCF7-PR cells. SA-β-galactosidase-positive cells increased by CDK2 siRNA (12.5 nM) or palbociclib (750 nM) and further increased by combination treatment. Green indicates the nuclear staining of β-galactosidase. *p*-values were calculated by student’s *t*-test after counting positive cells in 10 randomly chosen, non-overlapping fields. Data are presented as the mean ± S.E.M. Scale bar = 100 µm. Full length blots (**B**) are presented in Figure S5.

Therefore, we hypothesized that combined inhibition of CDK4/6 and CDK2 might inhibit overexpressed C-MYC and hTERT sequentially, thereby inducing senescence and preventing cancer progression. We treated MCF7 and MCF7-PR cells with palbociclib (IC_50_ concentration), CDK2 siRNA (12.5 nM), and a combination of those two for 72 h. As a result, overexpressed C-MYC and hTERT were significantly inhibited by the single treatment of CDK2 siRNA and further inhibited when combined with palbociclib treatment (*p* < 0.001) (Figure 5A). Because the phosphorylation site of C-MYC by CDK2 to stabilize C-MYC was previously reported to be ser62, we next analyzed the phosphorylation of C-MYC at ser62 following CDK2 inhibition. We found by western blot that the phospho-C-MYC (ser62) was significantly decreased by CDK2 siRNA and further decreased by the combination treatment (Figure 5B). Thus, we confirmed that C-MYC is phosphorylated at ser62 by CDK2, and consequently stabilized C-MYC eventually drives cells to escape senescence.

To further support the role of CDK2 in senescence, we performed a β-galactosidase assay using MCF7-PR cells. As expected, CDK2 siRNA-treated cells showed higher β-galactosidase expression compared with palbociclib-treated cells or the untreated control (*p* < 0.001). Moreover, combination-treated cells showed a significantly higher β-galactosidase expression compared with CDK2 siRNA-treated cells (*p* < 0.001) or palbociclib-treated cells (*p* < 0.001) (Figure 5C and Figure S4). These observations suggest that inhibition of CDK2 reduces C-MYC phosphorylation and inhibits the C-MYC target gene hTERT involved in senescence, leading to cancer cell death.

### 2.5. Combined Inhibition of CDK2 and CDK4/6 Overcomes Resistance to Palbociclib in a Palbociclib-Resistant Xenograft Model

**Figure 6:**
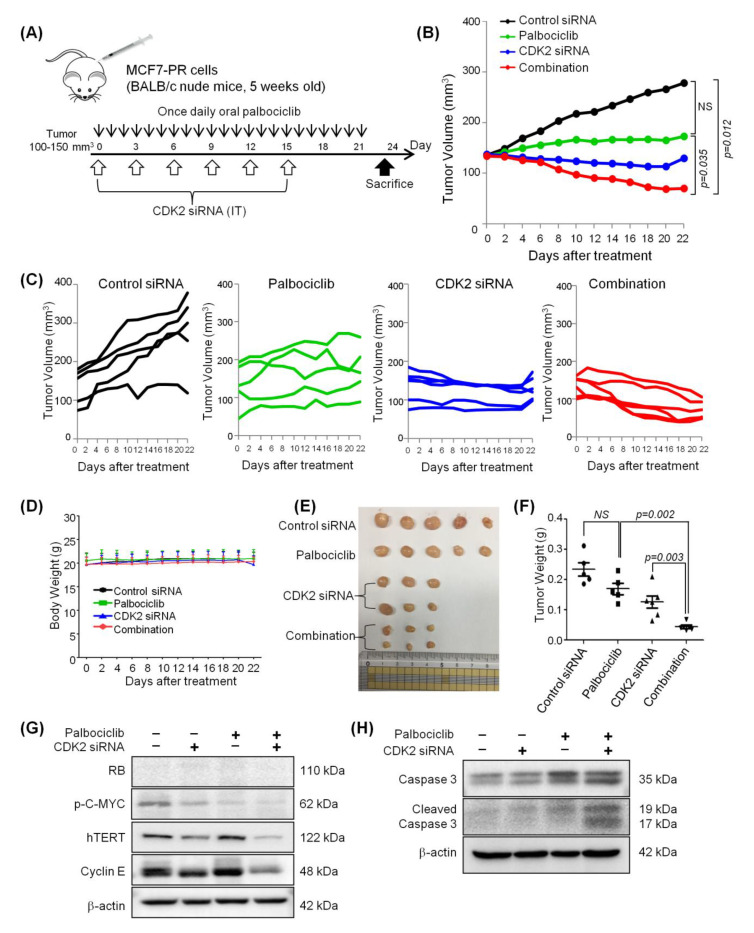
Combined treatment of CDK2 siRNA and palbociclib regresses palbociclib-resistant breast cancer synergistically in a xenograft model (**A**) Schemes for the in vivo experimental procedures to evaluate anticancer activities of CDK2 siRNA and palbociclib. (**B**,**C**) Mean (**B**) and individual (**C**) tumor growth curve of MCF7-PR cells treated with control siRNA, CDK2 siRNA, palbociclib, or a combination of palbociclib and CDK2 siRNA. Tumor volumes were monitored every 2–3 days. *p*-values were calculated by Student’s *t*-test on day 22 of treatment. Data are presented as the mean ± S.E.M. NS indicates not significant. (**D**) The body weights of mice indicated that the siRNA treatments and drug combinations did not cause any body weight loss. (**E**) The xenografted tumors were harvested on day 23 after drug treatment initiation. The pictures of the tumors were shown, and (**F**) the weights of the tumors were similar to those based on the calculated tumor volumes. *p*-values were calculated by student’s *t*-test. Data are presented as the mean ± S.E.M. NS indicates not significant. (**G**,**H**) Western blot using MCF7-PR xenograft after 23 days of treatment showed a greater suppression of phospho-C-MYC (ser62) and induction of cleaved caspase-3 in the combination group compared with palbociclib or CDK2 siRNA single treatment groups. Full length blots (**G**,**H**) are presented in Figure S5.

MCF7-PR xenograft mouse models were established to test in vivo tumor growth inhibition with CDK2 siRNA as a single agent and in combination with palbociclib (Figure 6A). Significant tumor regression was observed following treatment with CDK2 siRNA when combined with palbociclib compared with palbociclib treatment alone (*p* = 0.035) or the control siRNA treatment group (*p* = 0.012) (Figure 6B, C). Although the palbociclib treatment alone appeared to control growth, there was no significant regression of tumor growth (Figure 6B, C).

Importantly, none of the treatment caused weight loss, indicating a lack of generalized toxicity (Figure 6D). Finally, the mice were sacrificed on day 23 after drug treatment initiation, the tumors were excised (Figure 6E), and their weights were analyzed (Figure 6F). The results obtained were similar to those based on the calculated tumor volumes. Western blot analysis of the tumor tissue lysates showed greater inhibition of phospho-C-MYC (ser62) and hTERT in the combination group compared with palbociclib or CDK2 siRNA alone (Figure 6G), which confirmed again that C-MYC is involved in the ability of combined CDK2 and CDK4/6 inhibition to overcome acquired resistance to CDK4/6 inhibitor. Additionally, suppression of hTERT and induction of cleaved caspase-3 in the combination treatment group indicates that tumor regressed via senescence, leading to apoptosis [31] (Figure 6H).

### 2.6. High Cyclin E Expression Predicts Palbociclib Resistance and Poor Prognosis in HR-Positive Breast Cancer Patients

**Figure 7:**
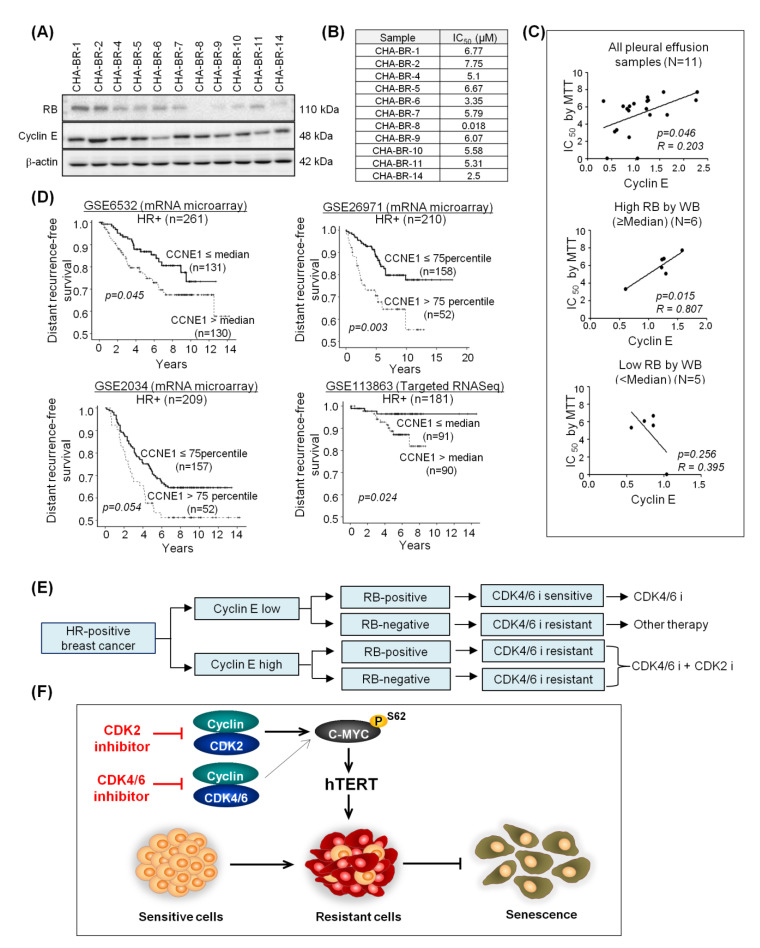
Pleural effusion analysis from HR-positive breast cancer patients supports the correlation between cyclin E overexpression and palbociclib resistance. (**A**) Western blot analysis of RB and cyclin E in pleural effusion cancer cells. (**B**) IC_50_ concentrations for the 6 days of palbociclib treatment, which were determined by the MTT assay. (**C**) The significant correlation of cyclin E overexpression and palbociclib resistance was observed in the analysis of all samples (*n* = 11). In the high RB group (≥median; *n* = 6), the correlation was more evident. However, palbociclib resistance was independent of cyclin E expression in the low RB group (<median; *n* = 5). (**D**) High CCNE1 expression predicts poor prognosis in HR-positive breast cancer patients. Kaplan-Meier curves indicate distant recurrence-free survival according to relative CCNE1 mRNA level from four independent public mRNA profiling data sets (**E**) Proposed biomarker-driven CDK inhibitor selection algorithm. (**F**) Schematic diagram of the mechanism of CDK2 and CDK4/6 mediated phosphorylation of C-MYC and subsequent proliferation of cancer cells via suppression of senescence. CDK2 and CDK4/6 inhibition induces senescence via inhibiting phospho-C-MYC (ser62) and sequentially hTERT, and thus, overcomes the acquired resistance to CDK4/6 inhibitors. The variation in the thickness of arrow demonstrates the efficacy to phosphorylate the C-MYC (ser62). Full length blots (**A**) are presented in Figure S5.

To investigate the clinical relevance of activated cyclin E-CDK2 signaling as a resistance biomarker and therapeutic target, we collected 11 pleural effusion samples from HR-positive breast cancer patients and correlated RB and cyclin E expression (Figure 7A) with palbociclib sensitivity (Figure 7B). We confirmed the significant correlation between cyclin E overexpression and palbociclib resistance in human samples (R = 0.203, *p* = 0.046) as demonstrated in the preclinical model. More intriguingly, this correlation was more evident in the high RB group (*n* = 6; R = 0.807, *p* = 0.015), but not in the low RB group (*n* = 5; R = 0.395, *p* = 0.256) (Figure 7C). This suggests that cyclin E overexpression plays a significant role in predicting resistance to CDK4/6 inhibitor in addition to low RB status. Furthermore, we investigated the impact of cyclin E overexpression on prognosis in four independent public mRNA expression data sets in HR-positive early breast cancer patients who did not receive CDK4/6 inhibitors (Table S4). CCNE1 overexpression was associated with a higher risk of distant recurrence as demonstrated by univariate analysis (Figure 7D). Multivariate analysis, adjusted by T and N stage, showed that cyclin E overexpression was a significant prognostic factor in HR-positive early breast cancer patients (Table S5). Taken together, the clinical relevance that cyclin E-CDK2 signaling could be a therapeutic target is further strengthened by our analysis of the public gene expression profiling data sets, even though they were not exposed to CDK4/6 inhibitors. Based on the association between cyclin E and resistance to CDK4/6 inhibitor mentioned above, we propose a biomarker-driven CDK inhibitor selection algorithm focusing on cyclin E (Figure 7E).

## 3. Discussion

The eventual progression of HR-positive breast cancer, even after treatment with CDK4/6 inhibitors, has limited the success of this therapy in the clinic, emphasizing the need to identify mechanisms of this acquired resistance. To explore the mechanisms of resistance to CDK4/6 inhibitor, we established reliable preclinical models of acquired palbociclib resistance, as demonstrated by the 10-fold increase in the IC_50_ of the drug, EMT transition, and cell cycle alteration. Our preclinical model confirmed that loss of RB [14,21,32] and overexpression of cyclin E [14,22], are associated with acquired resistance to palbociclib, as published in previous studies. Considering the wide use of CDK4/6 inhibitors in HR-positive breast cancer, our resistant model will be very valuable to develop drugs to overcome resistance. 

In addition to the loss of RB and cyclin E overexpression, there may be several other mechanisms involved in resistance to CDK4/6 inhibitor, as mentioned earlier. In particular, it has recently been reported that aberrant FGF2-FGFR1 signaling promoted the escape of cells from CDK4/6 inhibition by inducing cyclin D expression [33]. In another recent report, MDM2 amplification was associated with resistance to CDK4/6 inhibitor. In this study, an MDM2 antagonist was shown to induce p21expression, which augmented the activity of CDK4/6 inhibitors [34]. Of these several potential mechanisms of resistance, the activation of cyclin E-CDK2 signaling has been highlighted as one of the bypass mechanisms to escape CDK4/6 inhibition [35]. In our study, CCLE data analysis showed a significant correlation of cyclin E overexpression and palbociclib resistance. Our pleural effusion sample analysis from HR-positive breast cancer patients also supported this correlation. Furthermore, in line with the preclinical data, the recent PALOMA-3 translational study [22] that analyzed clinical samples, also confirmed cyclin E overexpression as the resistant mechanism. Clinical relevance that cyclin E-CDK2 signaling could be a therapeutic target is more strengthened by our analysis of the public gene expression profiling data sets from HR-positive breast cancer patients, even though they were not exposed to CDK4/6 inhibitors. Cyclin E forms a complex with CDK2 and activates its kinase function to promote the cell cycle progression of cells to the S phase [23]. Since CDK2 and CDK4/6 are both in the same class of kinase involved in cell cycle progression, we thought that CDK2 may be a potentially promising therapeutic target in CDK4/6 inhibitor-resistant cases, which motivated us to focus on the cyclin E-CDK2 pathway in this study.

The transcription factor C-MYC is an important oncogene that is frequently deregulated in human cancers [36]. The overexpression of C-MYC is associated with tumorigenesis and cell proliferation [37,38]. Moreover, the overexpression of C-MYC is involved in drug resistance in breast cancer [39,40]. Similar to previous observations, we also demonstrated that C-MYC was significantly overexpressed in our CDK4/6 inhibitor-resistant model. Mechanistically, CDK2 plays an important role in regulating C-MYC to suppress oncogene-induced senescence [41,42]. Precisely, CDK2 interacts and phosphorylates C-MYC at the promoter region of several genes involved in cellular senescence, such as p21, p16, Bmi-1, and hTERT [30]. This provides the mechanistic insight into how CDK2 and C-MYC are involved in senescence (Figure 7F).

Given that intact RB is required for the CDK4/6 inhibitor to have an effect, how the combined CDK2 and CDK4/6 inhibition synergizes in the absence of RB, reflecting the resistance to CDK4/6 inhibitor setting, remains unknown. The potential crosstalk between C-MYC and RB, which was demonstrated by simultaneous observation of RB loss and C-MYC overexpression in previous studies, [43,44] could explain how CDK2 inhibition may work even in the absence of RB, i.e., targeting CDK2 and CDK4/6 simultaneously can synergize to kill cancer cells via suppressing C-MYC and inducing senescence, independently of the RB status. This provides a novel synergistic mechanism how targeting both CDK2 and CDK4/6 overcomes resistance to CDK4/6 inhibitor.

Based on our results, we propose a biomarker-driven selection algorithm for CDK inhibitors focusing on RB and cyclin E. CDK4/6 inhibitors are the drug of choice for patients with low expressed cyclin E and intact RB. However, patients with overexpressed cyclin E exhibit resistance to CDK4/6 inhibitor, irrespective of the RB status. In such cases, the addition of a CDK2 inhibitor to CDK4/6 inhibitor treatment could overcome this resistance. Lastly, in RB-negative cases without cyclin E overexpression, which are resistant to both CDK4/6 and CDK2 inhibitors due to the lack of targets, targeted therapies other than CDK inhibitors should be given.

## 4. Materials and Methods 

### 4.1. Resistant Cell Line Establishment

Drug resistant cell lines were established by gradually increasing the concentration of drug starting from the concentration that was half of the maximal inhibitory concentration (IC_50_), which was 750 nM for MCF7 cells and 250 nM for T47D. Fresh media and media containing drug was replenished every 3 days. The drug concentration increased once the cells were able to proliferate freely in drug-containing media. The resistant cells were established after 7–9 months with almost a 10-fold increase in the IC_50_ concentration and named MCF7-PR and T47D-PR, indicating palbociclib-resistant MCF7 and T47D, respectively. Resistant cells were subsequently maintained in 1/5 of the IC_50_ concentration of palbociclib thereafter, and the drug was washed out for 48 h before experiments were performed.

### 4.2. Cancer Cell Line Encyclopedia (CCLE) Analysis

Cell line information, gene expression, and drug screening data were downloaded from the CCLE website (GSE36133) [24]. Using 38 breast cancer cell lines out of CCLE data, the expression of cell cycle specific genes was correlated with the palbociclib sensitivity, which was defined as an IC_50_ of ≤500 nM. A list of 38 breast cancer cell lines is given in Table S3.

### 4.3. Senescence Associated (SA)-β-Galactosidase Staining

Cells were seeded in six-well plates and treated with CDK2 siRNA and/or in combination with palbociclib, as described above. The cells were stained with SA-β-galactosidase 72 h after treatment using the SA-β-galactosidase staining kit (cat# 9860, Cell Signaling Technology, Boston, MA, USA) according to the manufacturers’ instructions. The cells were incubated with the staining solution at 37 °C (no CO_2_) overnight and washed twice with PBS. Blue-stained cells were identified as SA-β-galactosidase-positive cells by standard light microscopy. For each evaluation, 10 fields that were randomly chosen were counted, and the percentage of positively stained cells was calculated.

### 4.4. Animal Studies

Four weeks old BALB/c nude mice were purchased from Orient Bio Inc. (Seongnam, Korea). MCF7-PR cells mixed with matrigel (Corning, New York, NY, USA) were inoculated subcutaneously into the mammary fat pad of the mouse. Mice bearing tumor volume 100–150 mm^3^ were grouped and treated with control siRNA, palbociclib, hCDK2-siRNA and the combination of palbociclib and hCDK2 siRNA. Tumor growth was monitored three times a week, and tumor volume was calculated as (length^2^ × tumor width × 0.5). Further details are available in the Supplementary Methods.

### 4.5. Clinical Samples 

Pleural effusion samples were collected from HR-positive metastatic breast cancer patients. Each sample (50–200 mL) was first filtered through a 100 µM cell strainer and centrifuged at 400 g at 4 °C for 10 min, and the precipitated cells were washed with PBS. Mononuclear cells were isolated after Ficoll-paque Plus (d = 1.077 g/mL: cat#GE17-1440-02, GE Healthcare, Sigma-Aldrich, Inc., St. Louis, MO, USA) density gradient centrifugation at 400 g for 30 min. Cells were washed twice with PBS at 100 g for 10 min and resuspended with the culture media. Once cells were confluent, they were subcultured. Passage two or three cells were used for the cell viability assay and western blot analysis. The collection of clinical samples was approved by the Institutional Review Board (IRB) at the CHA Bundang Medical Center to collect patients’ pleural effusion samples (IRB number: 2016-03-037-012).

### 4.6. Public Gene Expression Profiling Data Sets in Breast Cancer Patients

To validate that CCNE1 mRNA expression level is associated with the prognosis in HR-positive breast cancer patients; we used four independent public mRNA expression data sets of curatively resected HR-positive early breast cancer (Table S4). Three data sets [GSE6532 [45], GSE26971 [46], GSE2034 [47]] are mRNA microarray data and one data set [GSE113863 [48]] is targeted mRNA sequencing data. Series matrix files which were already normalized by original authors, were downloaded for our analyses.

### 4.7. Study Approval

All animal procedures were performed according to the approved protocol by the Institutional Animal Care and Use Committee (IACUC) of CHA University (IACUC 180124). The collection of clinical samples was approved by the Institutional Review Board (IRB) at the CHA Bundang Medical Center to collect patients’ pleural effusion samples (IRB number: 2016-03-037-012). The written informed consent was received from participants prior to inclusion in the study.

Detailed Methods are in the Supplementary Materials.

## 5. Conclusions

Collectively, our study demonstrated the combined CDK2 and CDK4/6 inhibition overcomes cyclin E-associated resistance to CDK4/6 inhibitor by enhancing senescence, independently of the RB status. This is a novel mechanism behind synergism of targeting both CDK2 and CDK4/6. Our finding validated CDK2 as a promising therapeutic target. To date, there are no specific CDK2 inhibiting drugs available. Therefore, the development of a CDK2-specific kinase inhibitor is a rational approach for the treatment of breast cancers that are resistant to CDK4/6 inhibitor.

## Supplementary Materials

The following are available online at https://www.mdpi.com/2072-6694/12/12/3566/s1, Figure S1: Derivation and confirmation of HR-positive cells resistant to CDK4/6 inhibitors, Figure S2: Alteration of cell cycle and protein expression in palbociclib-resistant cells, Figure S3: CDK2 siRNA effectively inhibits CDK2, Figure S4: SA-β-gal staining in T47D-PR cells, Figure S5: PDF file. Un-cropped immunoblot images. Original images used to generate panels in Figure 2, Figure 3, Figure 5, Figure 6, Figure 7, Figures S1, S3 and S4, Table S1: Primary antibodies used for western blot or immunohistochemistry, Table S2: Primers used for qRT-PCR, Table S3: A list of 38 breast cancer cell lines in CCLE data base, Table S4: Patient characteristics in four public mRNA expression data sets, Table S5: Multivariate analysis of prognostic factors for distant recurrence-free survival in hormone receptor-positive breast cancer
