# Supplementary material for: Combined CDK2 and CDK4/6 Inhibition Overcomes Palbociclib Resistance in Breast Cancer by Enhancing Senescence
Source: Cancers (Basel). 2020 Nov 29;12(12):3566. doi: 10.3390/cancers12123566 (PMC7768442; doi:10.3390/cancers12123566)

Figure 2

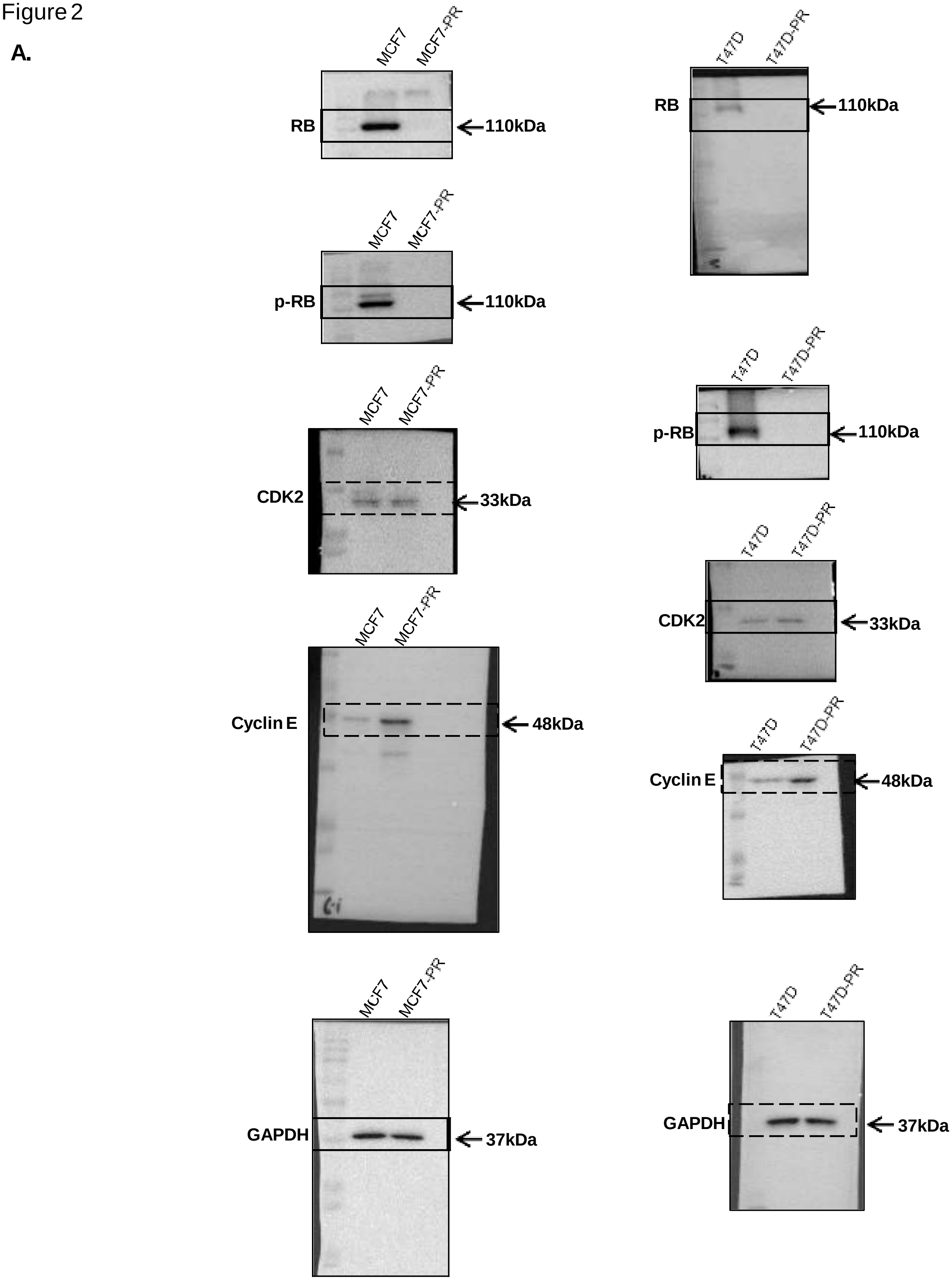
A.
MCF7
MCF7-PR
RB
110kDa
p-RB
110kDa
CDK2
33kDa
Cyclin E
48kDa
GAPDH
37kDa
T47D
T47D-PR
RB
110kDa
p-RB
110kDa
CDK2
33kDa
Cyclin E
48kDa
GAPDH
37kDa

Figure 3

D.

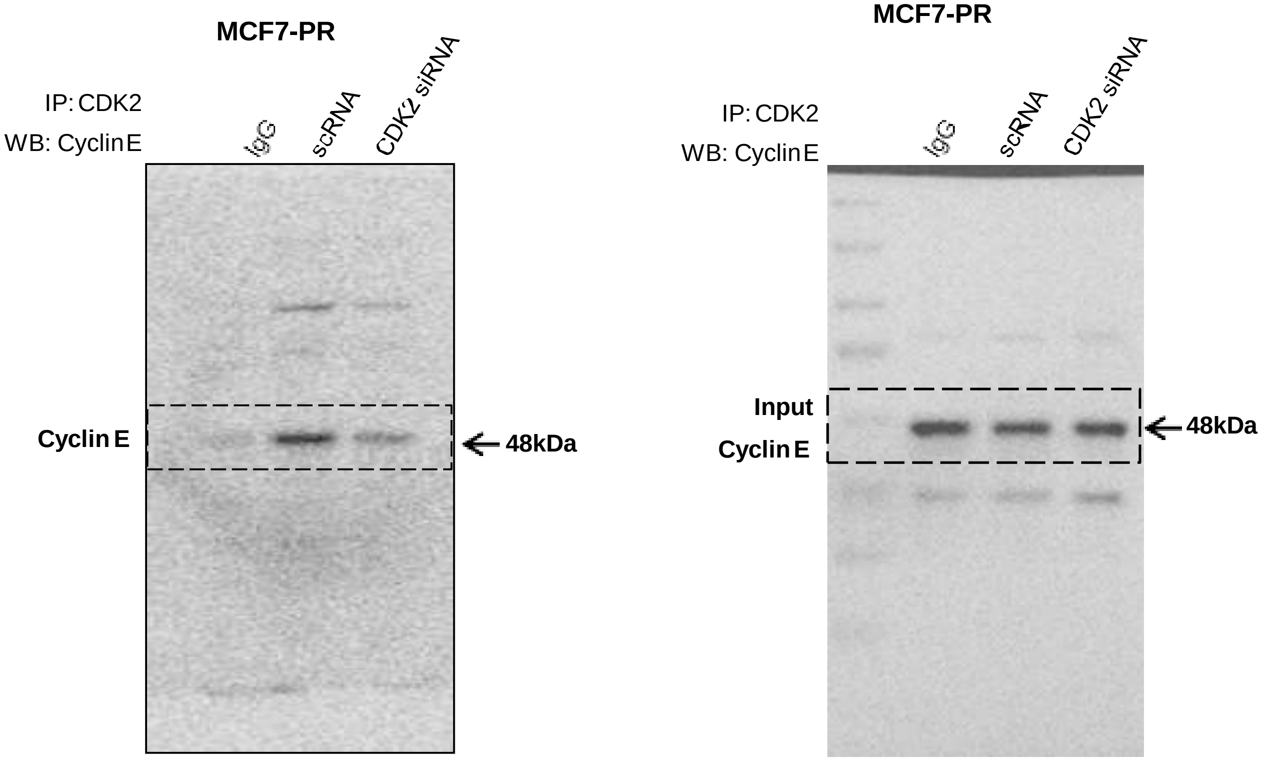
MCF7-PR
IP: CDK2
WB: Cyclin E
IgG
scRNA
CDK2 siRNA
Cyclin E
48kDa
MCF7-PR
IP: CDK2
WB: Cyclin E
IgG
scRNA
CDK2 siRNA
Input
Cyclin E
48kDa

E.

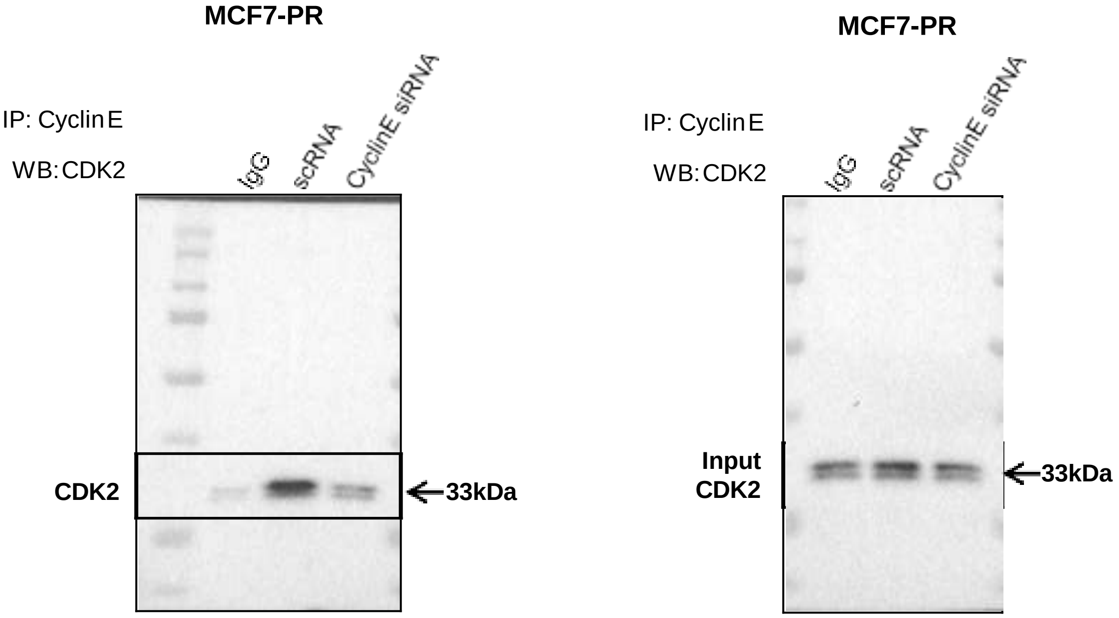
MCF7-PR
IP: Cyclin E
WB: CDK2
IgG
scRNA
CyclinE siRNA
CDK2
33kDa
MCF7-PR
IP: Cyclin E
WB: CDK2
IgG
scRNA
CyclinE siRNA
Input
CDK2
33kDa

Figure 5

B.

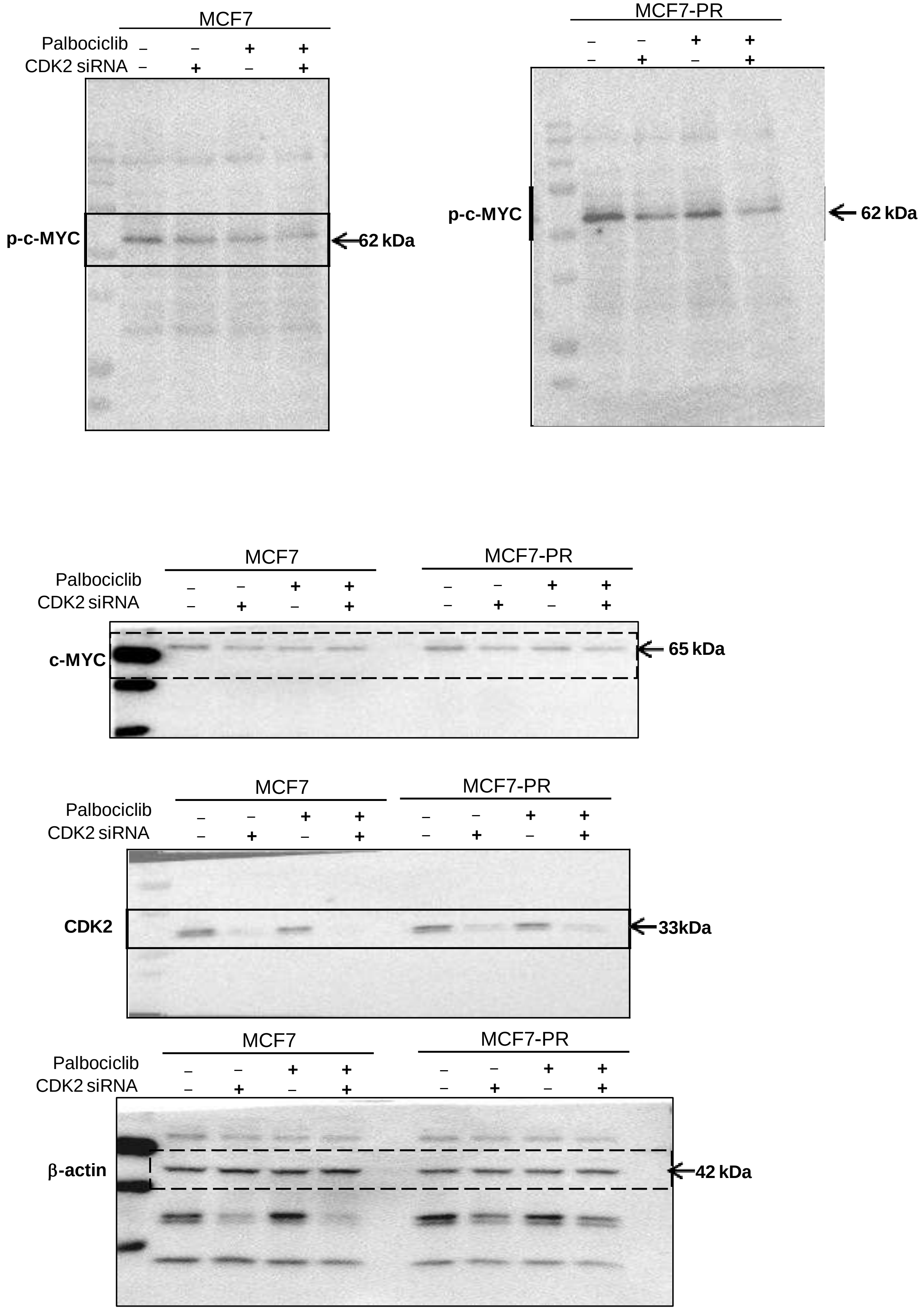
MCF7
Palbociclib
CDK2 siRNA
p-c-MYC
62 kDa
MCF7-PR
p-c-MYC
62 kDa
MCF7
MCF7-PR
Palbociclib
CDK2 siRNA
c-MYC
65 kDa
MCF7
MCF7-PR
Palbociclib
CDK2 siRNA
CDK2
33kDa
MCF7
MCF7-PR
Palbociclib
CDK2 siRNA
β-actin
42 kDa

Figure 6.

G.

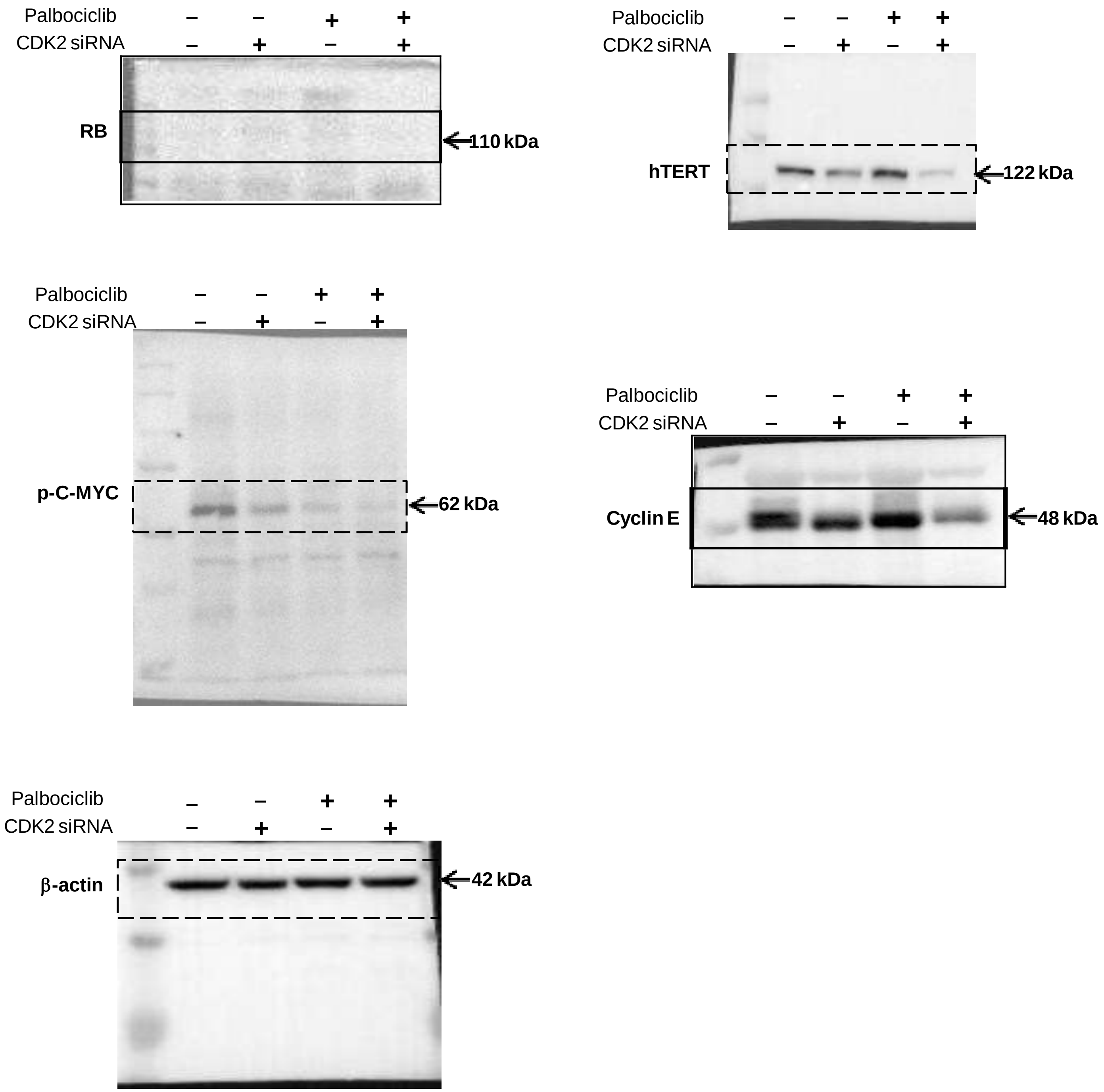

Palbociclib
CDK2 siRNA
RB
110 kDa
hTERT
122 kDa
p-C-MYC
62 kDa
Cyclin E
48 kDa
β-actin
42 kDa

Figure 6.

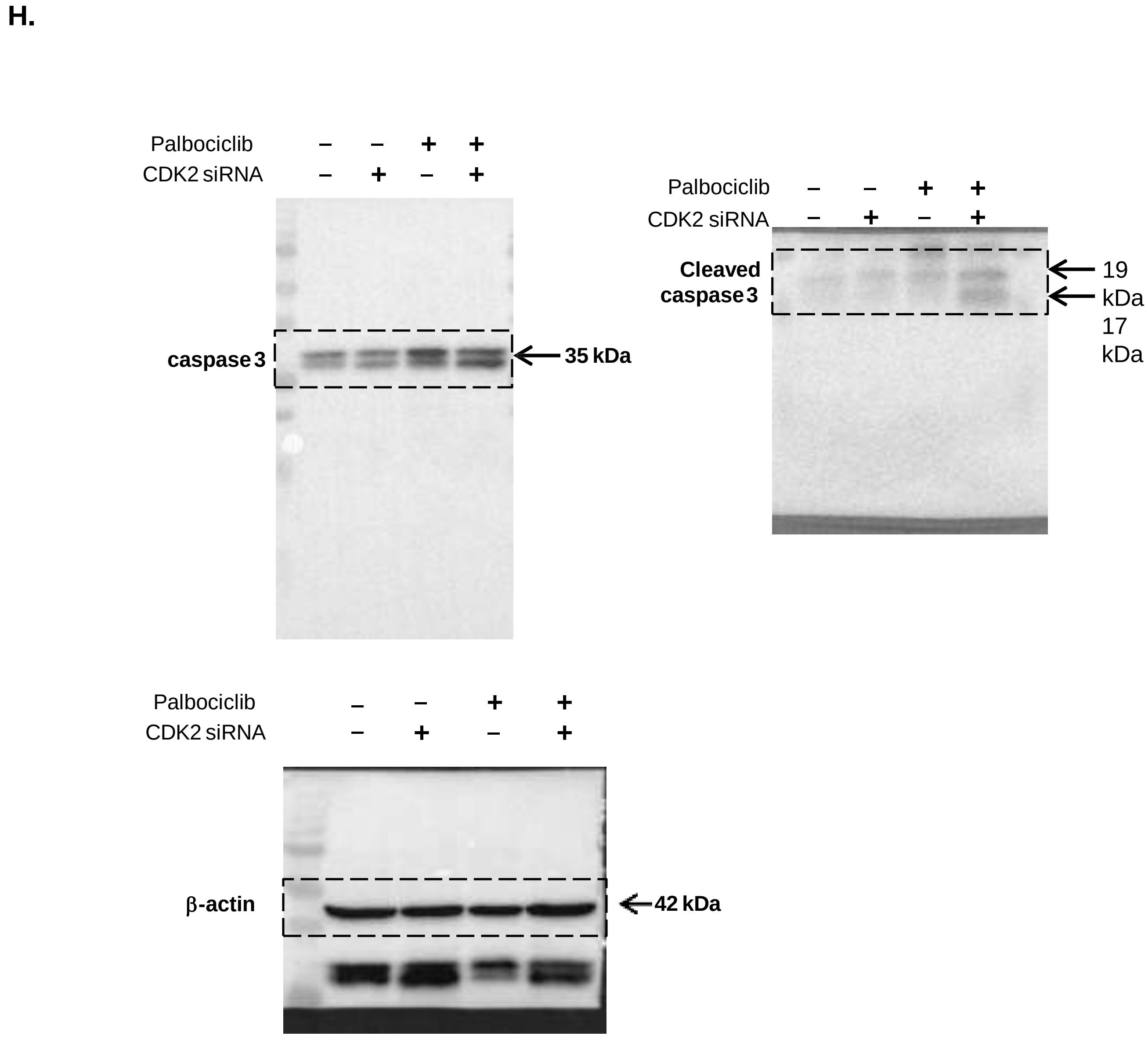

H.
Palbociclib – – + +
CDK2 siRNA – + – +
caspase 3
35 kDa
Palbociclib – – + +
CDK2 siRNA – + – +
Cleaved caspase 3
19 kDa
17 kDa
Palbociclib – – + +
CDK2 siRNA – + – +
β-actin
42 kDa

Figure 7

**A.**

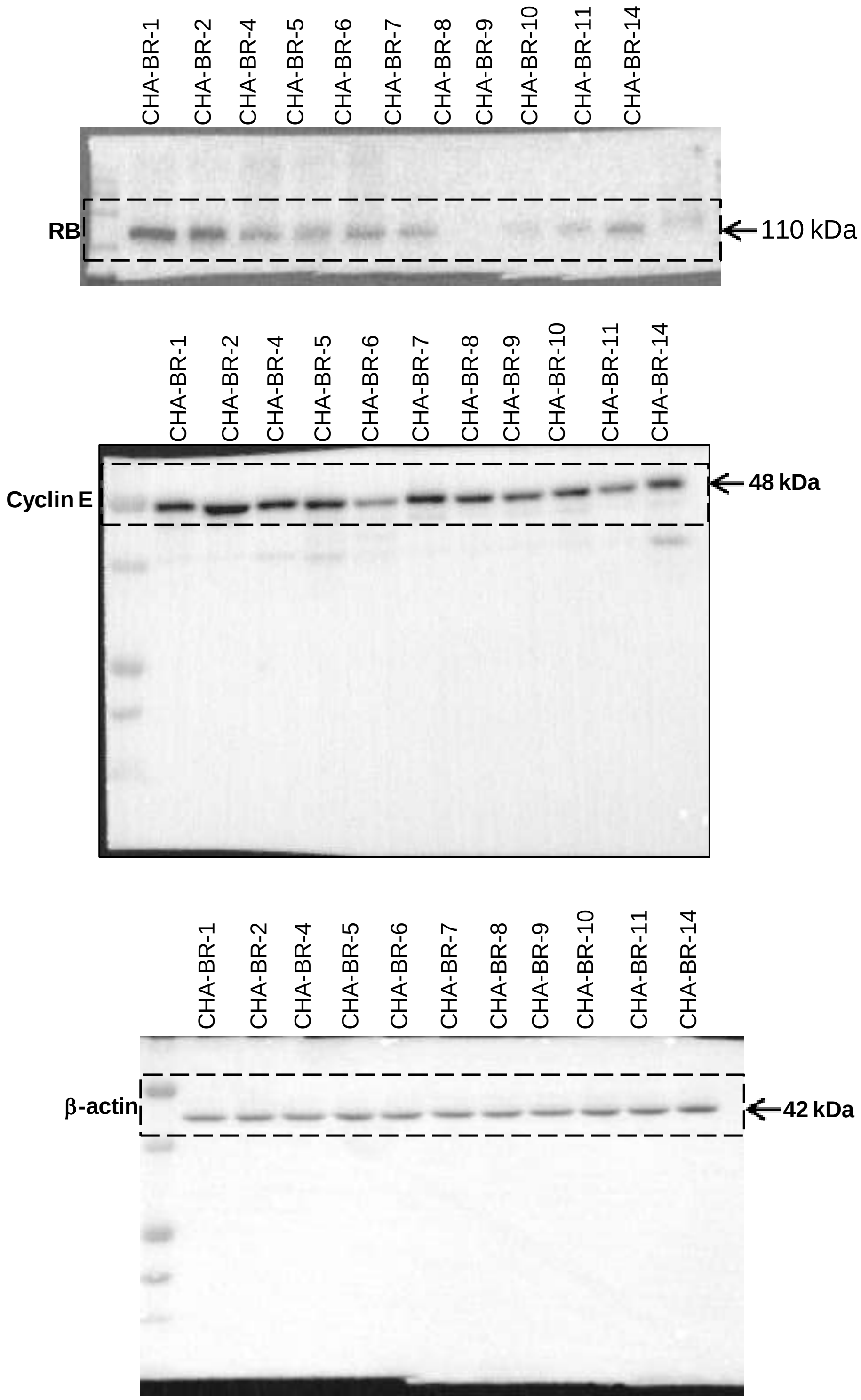
CHA-BR-1
CHA-BR-2
CHA-BR-4
CHA-BR-5
CHA-BR-6
CHA-BR-7
CHA-BR-8
CHA-BR-9
CHA-BR-10
CHA-BR-11
CHA-BR-14
RB
110 kDa
Cyclin E
48 kDa
β-actin
42 kDa

Supplementary Figure1

C.

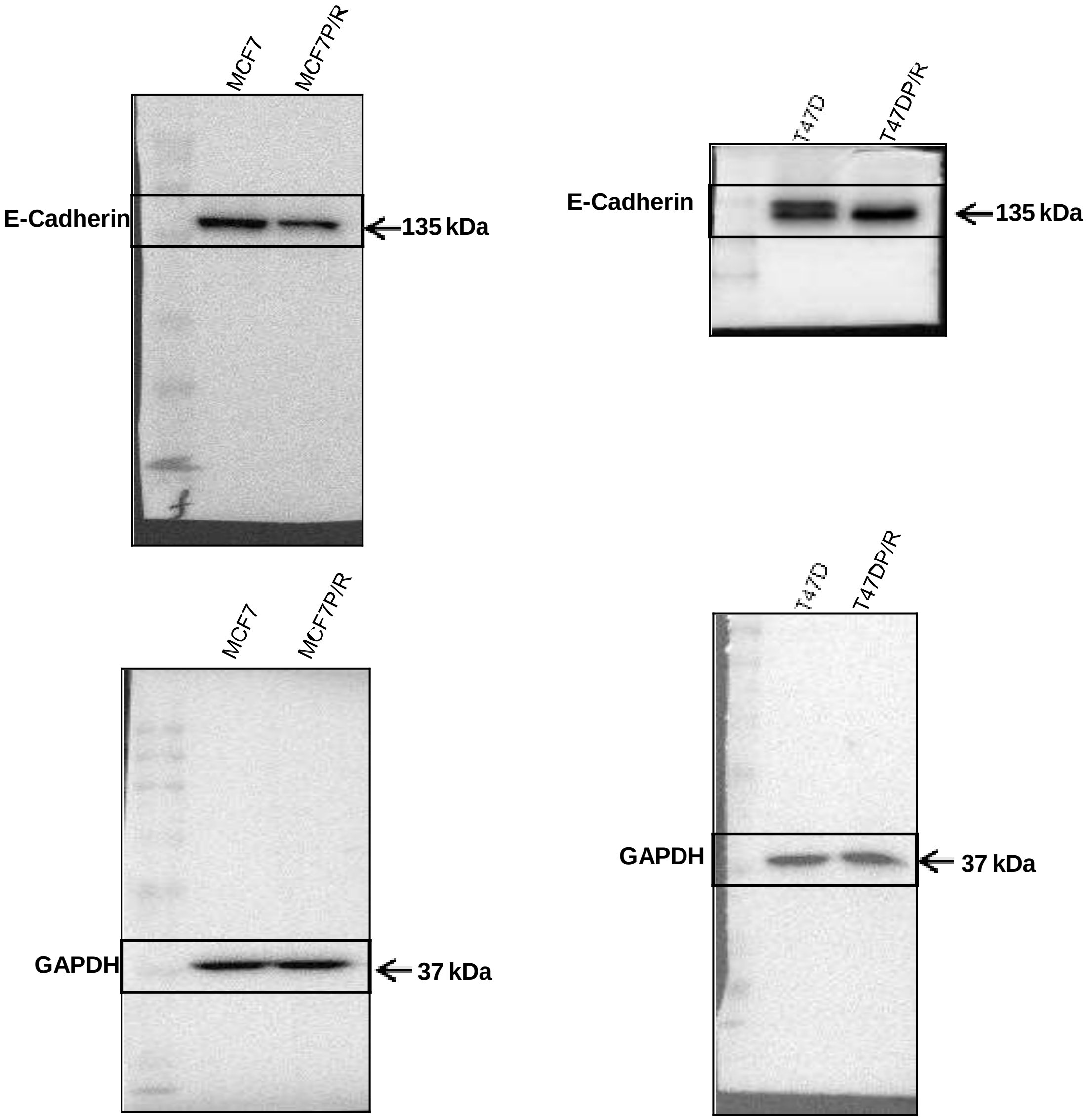

## Supplementary Figure 3

**MCF7-PR**

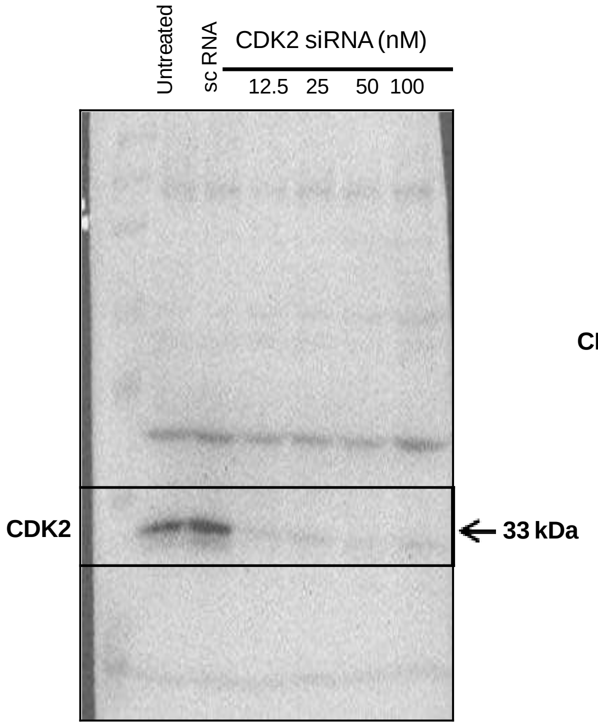


**T47D-PR**

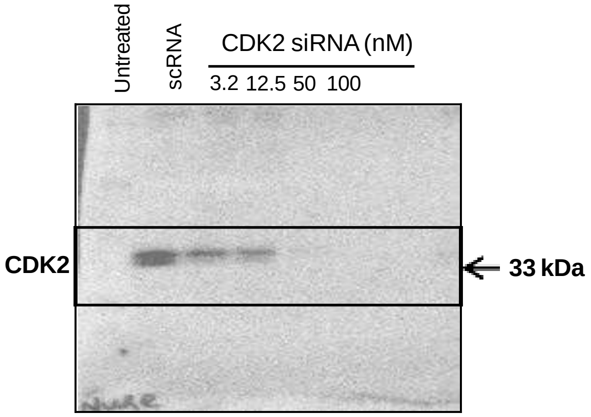


**MCF7-PR**

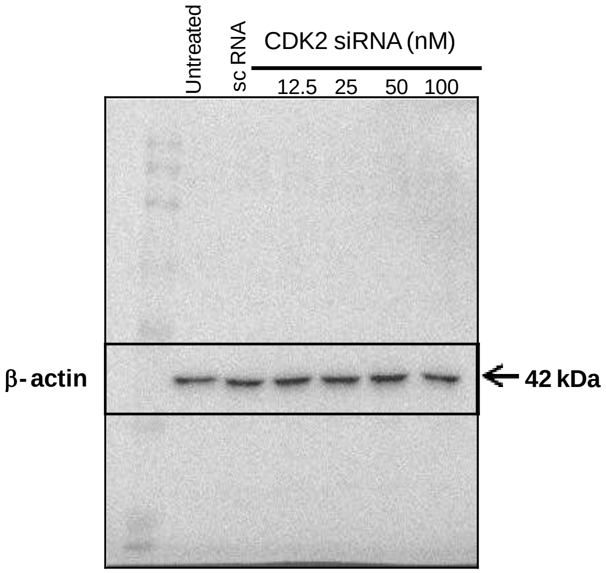

**Supplementary Figure 4**

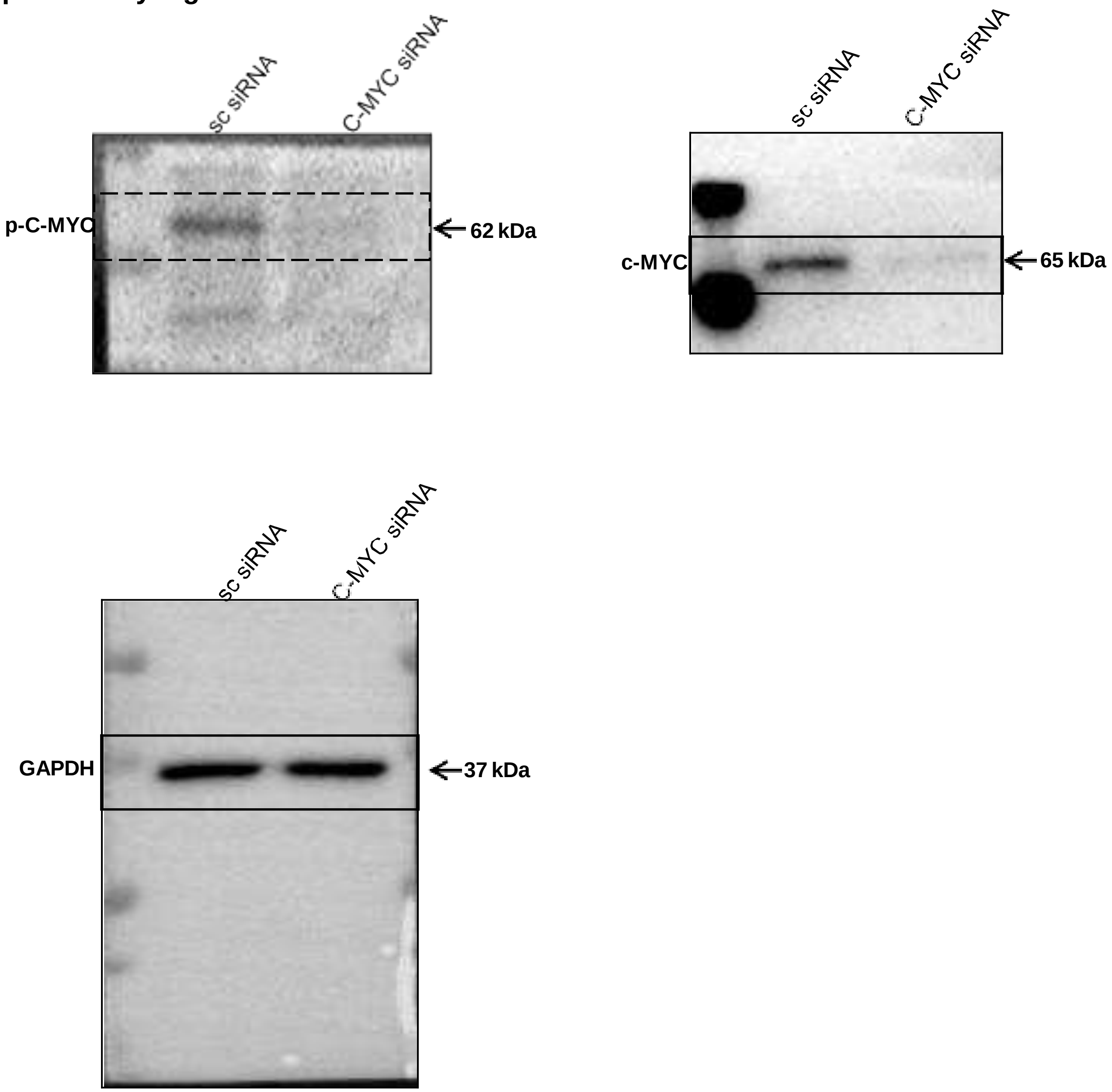

Supplement: Supplementary file 1 [file cancers-12-03566-s001.zip › cancers-1008945-Figure S5_Uncropped immunoblot.corrected1.pdf]
