# Supplementary material for: Combined CDK2 and CDK4/6 Inhibition Overcomes Palbociclib Resistance in Breast Cancer by Enhancing Senescence
Source: Cancers (Basel). 2020 Nov 29;12(12):3566. doi: 10.3390/cancers12123566 (PMC7768442; doi:10.3390/cancers12123566)
Supplement: Supplementary file 1 [file cancers-12-03566-s001.zip › cancers-1008945-Supplementary files.CLEAN.pdf]

*cancers*

MDPI

*Article*

# Combined CDK2 and CDK4/6 Inhibition Overcomes Palbociclib Resistance in Breast Cancer by Enhancing Senescence

**Kamal Pandey [1,3], Nahee Park [1], Kyung-Soon Park [3], Jin Hur [1,3], Yong Bin Cho [1], Minsil Kang [1] Hee-Jung An [2], Sewha Kim [2], Sohyun Hwang [2,3] and Yong Wha Moon [1,*]**

[1] Hematology and Oncology, Department of Internal Medicine, CHA Bundang Medical Center, CHA University, Seongnam,13488, Korea; pkamal@chauniv.ac.kr (K.P.); skgml0413@naver.com (N.P.); hurjinz@naver.com (J.H.); ybyoungbin@naver.com (Y.B.C.); rbtnr1@naver.com (M.K.)

[2] Department of Pathology, CHA Bundang Medical Center, CHA University, Seongnam,13488, Korea; hjahn@cha.ac.kr (H.-J.A.); sewhakim@chamc.co.kr (S.K.); blissfulwin@cha.ac.kr (S.H.)

[3] Department of Biomedical Science, CHA University, Seongnam,13488, Korea; kspark@cha.ac.kr

* Correspondence: ymoon@cha.ac.kr; Tel.: +82-10-2825-6357; Fax: +82-31-780-3929

**Supplementary figures:**

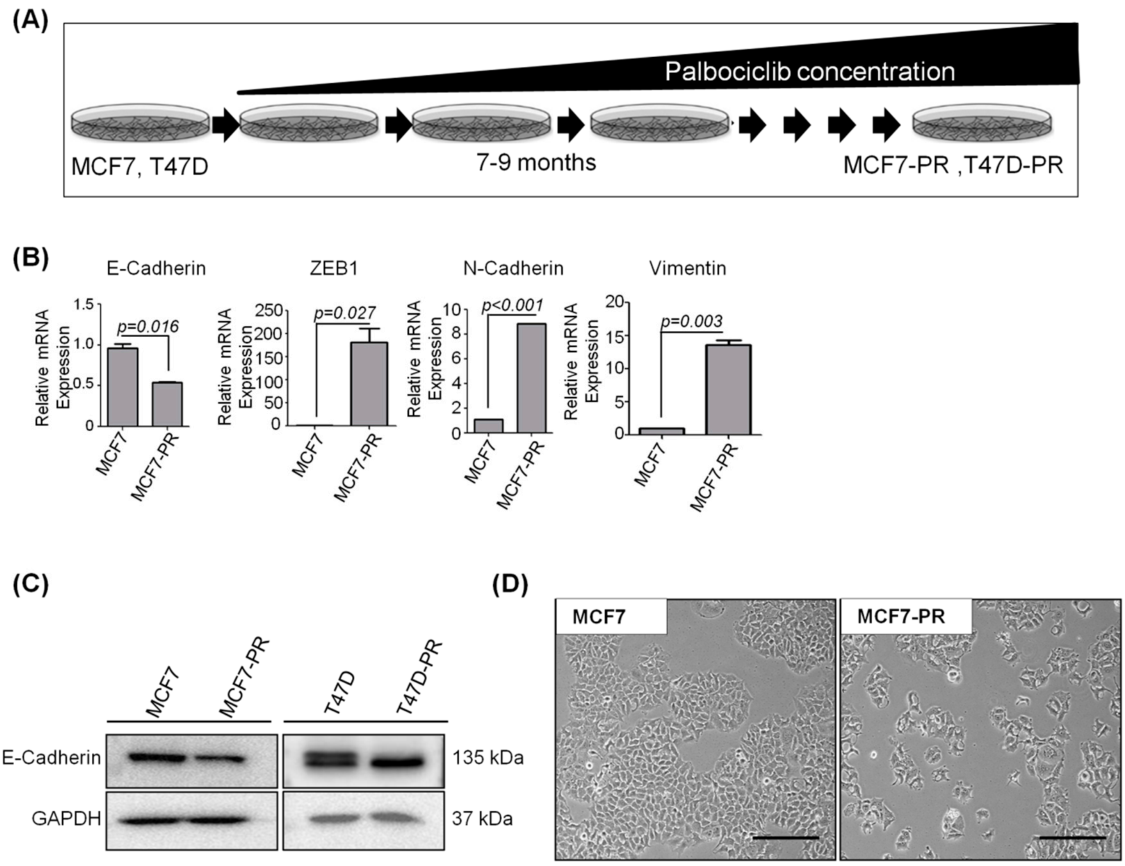


**Supplementary Figure S1.** Derivation and confirmation of HR-positive cells resistant to CDK4/6 inhibitors **(A)** Schematic diagram showing the generation of palbociclib-resistant cells. MCF7 and T47D cells were continuously treated with a gradually increasing concentration of palbociclib for 7-9 months and named as MCF7-PR and T47D-PR, respectively. **(B)** Palbociclib-sensitive and resistant cells were compared for the expression of EMT markers. qRT-PCR analysis of the indicated genes

was performed. *P*-values were calculated by student's *t*-test. Data are presented as the mean ± S.E.M. of triplicate experiments. **(C)** Western blot analysis showing the decrease of the epithelial marker E-cadherin in the palbociclib-resistant cells compared with their sensitive counterparts. **(D)** Images showing the morphological changes of MCF7-PR (mesenchymal) compared with MCF7. Scale bars = 100 μm. Full length blots **(C)** are presented in Figure S5.

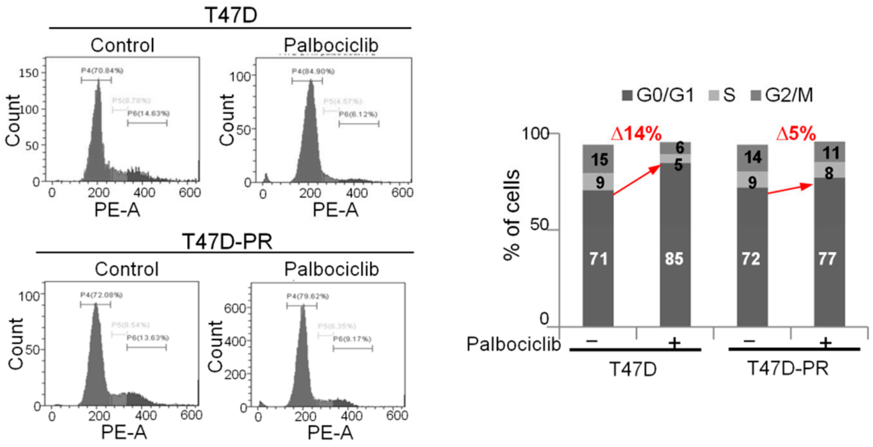


**Supplementary Figure S2.** Alteration of cell cycle and protein expression in palbociclib-resistant cells. Similarly to MCF7 and MCF7-PR, T47D cells were significantly arrested at the G1 phase by palbociclib, however, T47D-PR cells were not blocked at G1 by palbociclib (260 nM).

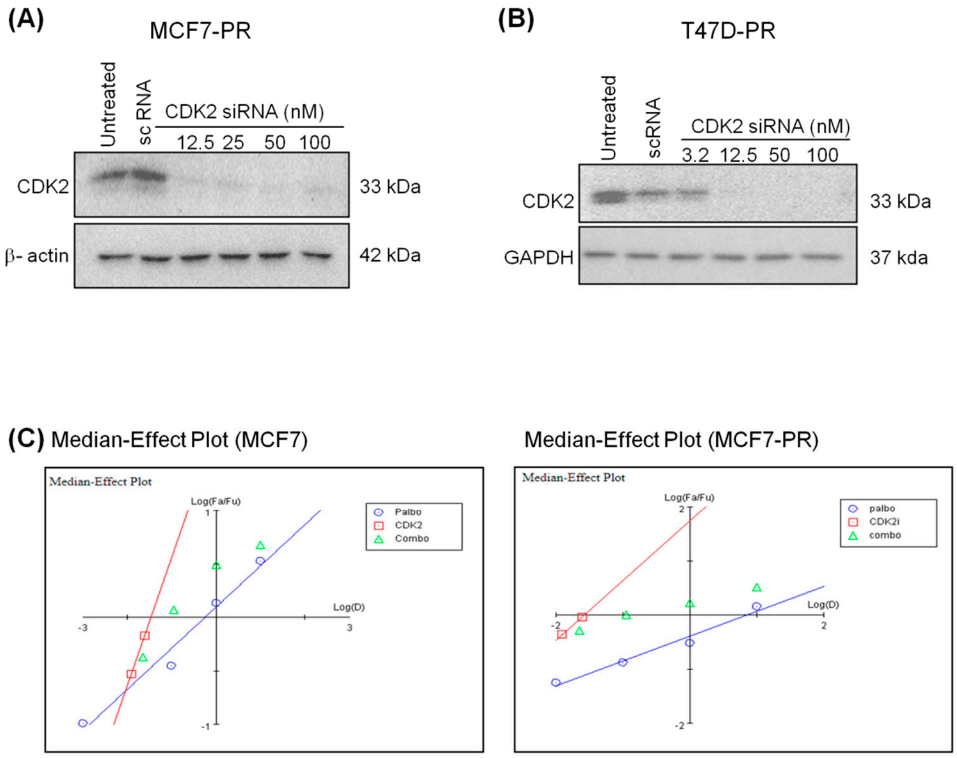


**Supplementary Figure S3.** CDK2 siRNA effectively inhibits CDK2. (**A**) MCF7-PR and **(B)** T47D-PR cells were treated with various concentrations of CDK2 siRNA for 48 hours. The complete inhibition of CDK2 was observed with 12.5 nM and higher concentrations**. (C)** Median-effect plot of MCF7 and MCF7-PR. Full length blots **(A, B)** are presented in Figure S5.

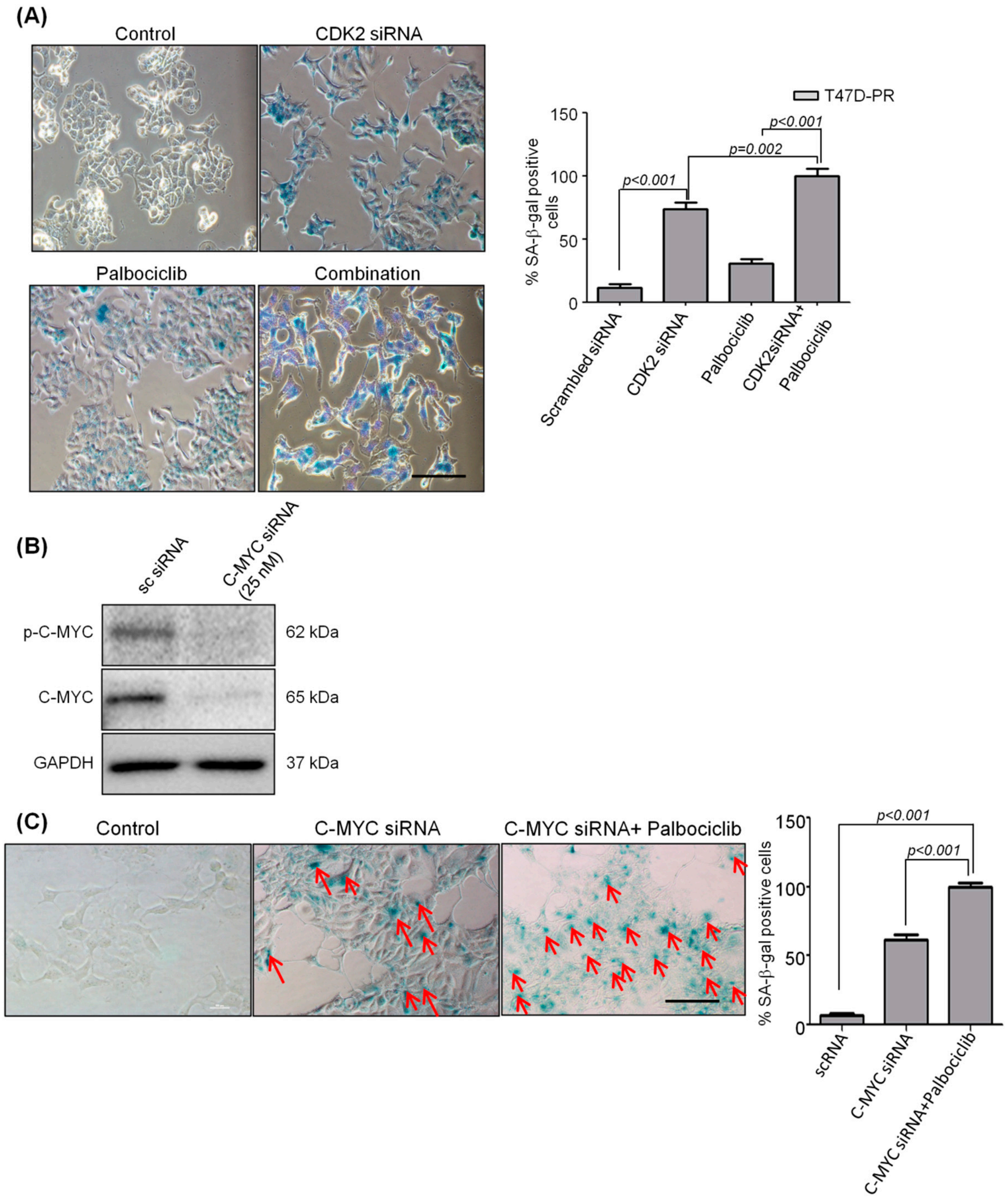


**Supplementary Figure S4.** SA-β-gal staining in T47D-PR cells. (A) In T47D-PR cells, SA-β-gal-positive cells were increased by CDK2 siRNA (12.5 nM) or palbociclib (260 nM) and further increased by combination treatment, which was similar to MCF7-PR cells. Green indicates the nuclear staining of β-galactosidase. *P* values were calculated by *t*-test after counting positive cells in 10 randomly chosen, non-overlapping fields. Data are presented as the mean ± S.E.M. Scale bar = 100 μm. (B) MCF7-PR cells were treated with C-MYC siRNA for 48 hours. The complete inhibition of phospho-C-MYC (ser62) and C-MYC was observed with 25 nM concentration. (C) In MCF7-PR cells, SA-β-gal-positive cells were increased by C-MYC siRNA (25 nM) and further increased by combination treatment with palbociclib. Green indicates the nuclear staining of β-galactosidase. *P* values were calculated by *t*-test after counting positive cells in 10 randomly chosen, non-overlapping fields. Data are presented as the mean ± S.E.M. Scale bar = 100 μm. Full length blots **(B)** are presented in Figure S5.

## Supplementary Methods

### Drugs

Palbociclib (PD-332991) was provided by Pfizer Inc. (North Peapack, NJ 07977, USA). Abemaciclib and ribociclib were purchased from ChemScene (NJ 08852, USA). Palbociclib was dissolved in water, whereas all other drugs were reconstituted in dimethyl sulfoxide (DMSO) (Sigma-Aldrich).

### Cell culture

All breast cancer cell lines were purchased from the American Type Culture Collection (Manassas, VA, USA) and maintained in a humidified atmosphere with 5% CO2 at 37°C. MCF7 and T47D cells were cultured in RPMI 1640 medium (Welgene Inc., Deagu, Korea) supplemented with 10% heat-inactivated fetal bovine serum (cat# S 001-01; FBS, Welgene Inc.) and 1% 100X penicillin/streptomycin solution (Welgene Inc.).

### Cell viability assay

The MTT assay was used to access the viability of the cells. Cells growing in a logarithmic phase were seeded at the density of 500-1000 cells per well in 96-well plates. Once the cells adhered to the dish 24 hours after plating, different concentrations of palbociclib and a fixed concentration of CDK2 siRNA were added to the cells and incubated for 24, 48, or 72 hours or for up to 6 days at 37°C. MTT solution (5 mg/ml) was added into each well, and 4 hours later, the medium was discarded. DMSO (100 μl) was added into the wells to dissolve the formazan product from the metabolism of MTT. Finally, the optical density was measured at 540 nm using a Microplate Reader (Multiskan GO Microplate Spectrophotometer). $IC_{50}$ and Combination Index (CI) values were analyzed using CompuSyn software (ComboSyn, Inc., NJ, USA).

### Western blot and co-immunoprecipitation

Cells were lysed in RIPA lysis buffer (cat# # 89901, Thermo Scientific) supplemented with a protease inhibitor cocktail (cat# 11873580001, Roche) and phosphatase inhibitor (cat# 1862495, Thermo Scientific). Total protein lysates were separated by SDS-PAGE and electrotransferred onto polyvinylidene difluoride (PVDF) membranes. Membranes were blocked for 1 hour in Tris-buffered saline (TBS) containing 5% milk and 0.1% Tween 20 at room temperature. All primary antibodies were incubated overnight at 4°C, followed by incubation with a secondary antibody conjugated with horseradish peroxidase for 1 hour at room temperature. Detection was performed with a Super Signal Chemiluminescent reagent according to the manufacturers' protocol (cat#32209, ThermoFisher Scientific, Waltham, MA USA).

For the co-immunoprecipitation assay, MCF7-PR cells were lysed in ice-cold NETN lysis buffer [250 mM NaCl, pH 8.0 5 mM Tris-HCL, 5 mM EDTA, and 0.5% NP-40 (IGEPAL CA-630, Sigma-Aldrich) supplemented with protease and phosphatase inhibitor cocktails]. Total protein lysates (500 μg) were then separately immunoprecipitated with anti-CDK2 (1:50) and anti-cyclin E (2 μg) antibodies overnight at 4°C in the presence of 50 μL of protein A/G agarose beads (cat#20421, ThermoFisher Scientific, Waltham, MA USA). Normal mouse IgG (cat# sc-2025, Santa Cruz Biotechnology) and rabbit IgG isotypes (cat#3900, Cell Signalling Technology) were used as internal

controls. Immunoblotting detection was completed, as described above. A list of antibodies is given in Supplementary Table S1.

**Quantitative real-time PCR (qRT-PCR)**

Total RNA was isolated from cells using TRIzol (Life Technologies). Total RNA was reverse transcribed to complementary DNA (cDNA) using the Takara prime script 1$^{st}$ strand cDNA synthesis kit (cat# 6110A, Takara Bio Inc). The quantitative real-time polymerase chain reaction was performed using an ABI StepOne Real-time PCR System (Applied Biosystems, Warrington, UK) and a Power-up SYBR Green Master Mix (cat#A25741, ThermoFisher scientific). Double-stranded DNA dissociation was assessed by melting curve analysis, and β-actin was used as the endogenous control. Gene expression levels were calculated and analyzed using the $2^{-\Delta\Delta ct}$ method. Primers were obtained from Macrogen (Macrogen, Inc. Korea). A list of primers is given in Supplementary Table S2.

**siRNA transfection**

Cells were seeded at a density of $3 \times 10^5$ cells/well were in six-well plates. Lipofectamine RNAiMAX Reagent (cat# 13778-030, Invitrogen) was used for siRNA transfections according to the manufacturers' instructions. siRNAs and lipofectamine were separately diluted in Opti-MEM® I Reduced-Serum Medium (cat#31985070, ThermoFisher Scientific) according to the manufacturers' procedure. Subsequently, the mixtures were added to the wells containing cells. Furthermore, the cells treated with only lipofectamine and scrambled siRNA were used as the siRNA blank control. The cells were then incubated for 24 to 72 hours at 37°C in a humidified CO2 incubator. After transfection, qRT-PCR and western blot analyses were performed to assess the effect of siRNA on gene silencing.

**Gene expression microarray analyses**

RNA samples were extracted using TRIzol reagent (Life Technologies, Carlsbad, CA, USA). The Affymetrix Whole Transcript Expression array process was executed according to the manufacturers' protocol (GeneChip Whole Transcript PLUS reagent Kit). For the quality control, RNA purity and integrity were evaluated by the OD 260/280 ratio and analyzed using an Agilent 2100 Bioanalyzer (Agilent Technologies, Palo Alto, USA). cDNA was synthesized using the GeneChip WT (Whole Transcript) Amplification kit, as described by the manufacturer. The sense cDNA was then fragmented and biotin labeled with TdT (terminal deoxynucleotidyl transferase) using the GeneChip WT Terminal labeling kit. Approximately 5.5 μg of labeled DNA target was hybridized to the Affymetrix GeneChip Human 2.0 ST Array at 45°C for 16 hours. Hybridized arrays were washed, stained on a GeneChip Fluidics Station 450, and scanned on a GCS3000 Scanner (Affymetrix). Signal values were computed using the Affymetrix® GeneChip™ Command Console software. Only genes that were upregulated or downregulated by 2-fold were considered meaningful.

**Fluorescence-activated cell sorting analysis (FACS) for cell cycle**

MCF7 and MCF7-PR cells ($3 \times 10^5$) were grown in 6 well plates in RPMI-1640 medium supplemented with 10% fetal bovine serum at 37 °C. Cells were treated with the $IC_{50}$ concentration of palbociclib for 48 hours. Cells were harvested and fixed overnight in 70% ethanol at -20°C. The cells were washed twice with ice-cold PBS, rehydrated, re-suspended in RNase A (100 µg/ml), and stained with propidium iodide (50 µg/ml) in PBS. The stained cells were incubated in the dark at room temperature for 20 minutes, and analyzed by flow cytometry (Beckman Coulter Cytoflex, Indianapolis, USA). Approximately 10,000 cells/sample were analyzed, and the percentage of cells in each phase of the cell cycle was determined.

**Immunohistochemistry of cell blocks**

MCF7 and MCF7-PR cells were collected by trypsinization. Following centrifugation, the pellets were kept at the center of the plate and surrounded with 1.5% agarose gel. When the gel solidified, the area of gel containing the cell pellet was cut, incubated in 4% PFA for fixation, and sent to the histology lab to prepare paraffin blocks.

For immunohistochemical staining, cell blocks were sectioned with a microtome to generate 5 µM sections, which were placed on slides. After deparaffinization, citrate-based antigen retrieval was performed, followed by blocking with a protein block serum-free medium (cat# X0909; DAKO, Carpinteria, CA) for 30 min at room temperature. The slides were then incubated with anti-RB (cat# 9309, cell signaling technology) or anti-cyclin E (cat# sc-248, Santa Cruz Biotechnology, Inc) primary antibodies overnight at 4°C in a humid chamber. The slides were washed 3 times with PBS and incubated with a secondary antibody for 1 hour at room temperature. Antigen-antibody complexes were detected by the avidin-biotin-peroxidase method, using 3, 3′-diaminobenzidine as a chromogenic substrate (cat# 1855920, Thermo Scientific). Finally, the slides were counterstained with hematoxylin (cat# S3309, DAKO, Carpinteria, CA). Coverslips were mounted after dehydration of the sections using permanent mounting medium (cat# C1795, Sigma-Aldrich) and then examined under a light microscope.

**Animal studies**

All mice were housed in a specific pathogen-free animal facility with a controlled temperature of 24 ± 3°C and a light/dark cycle of 12 hours with unlimited access to food and water at CHA University (Seongnam, Korea). Animals were acclimatized to the environment for at least 1 week prior to the experiment. All animal procedures were performed according to the approved protocol by the Institutional Animal Care and Use Committee (IACUC) of CHA University. A total of $1 \times 10^7$ MCF7-PR cells in 0.1 ml of PBS containing a 50%(V/V) Matrigel solution (corning, NY, USA) were inoculated subcutaneously into the mammary fat pad of the mouse. Estrogen valerate (3 µg/mouse) was subcutaneously injected every week as an estrogen supplement. When the tumor volume reached 100–150 $mm^3$, the animals were randomized into four groups. Each treatment group consisted of five or six mice. Mice were treated with control siRNA (group 1), palbociclib 100 mg/kg (group 2), 5 µg of hCDK2-siRNA (group 3), hCDK2- siRNA with palbociclib (group 4). siRNAs were formulated with *in vivo*-jetPEI (cat# 201-10G, Polyplus Transfection) according to the manufacturers' instructions using an N/P ratio of 6 and were injected intratumorally every 3 days for 6 times. Palbociclib (100 mg/kg) was injected by oral gavage once daily for 21 days. Tumor sizes

were measured using a Vernier caliper three times a week and calculated by the formula tumor length$^2$ × tumor width × 0.5. Additionally, bodyweight was assessed three times a week. After 23 days, all mice were sacrificed according to the animal experimental guidelines, and the xenografted tumors were excised and preserved for further analysis.

**Statistical analysis**

Student's *t*-test was performed to compare two groups in qRT–PCR, cell viability assay and SA-β-galactosidase assay whereas, western blot data were analyzed using paired *t*-test. The correlations between gene expression and palbociclib sensitivity in the CCLE data were analyzed using the Pearson correlation coefficient. Distant recurrence-free survival (DRFS) was defined as the time from curative surgery to recurrence in distant organs or the last date at which the patient was known to be free of distant recurrence (censoring time). DRFS was measured using the Kaplan-Meier estimator. In the public gene expression profiling data sets, to divide patients into two groups of high or low CCLE1 mRNA expression, an optimal cut-off score was selected as the quartile with the minimum log rank *P*-value in DRFS analysis. The statistical significance of comparisons of survival curves were calculated by log-rank test. Multivariate analysis for prognostic factors was performed by the Cox proportional hazards regression model. All statistical analyses were performed using SPSS version 19.0 (IBM SPSS statistics 19.0, NY, USA), except for Pearson correlation coefficient, which was performed using GraphPad Prism (version 5.01; GraphPad Software, Inc.). All *P*-values were two-sided, and *P*-values of less than 0.05 were considered significant

**Supplementary Tables**

Supplementary Table S1. Primary antibodies used for western blot or immunohistochemistry

| Antibody | Host species | Dilution | Company (Catalog#) |
|---|---|---|---|
| RB | Mouse | 1:1000 | 9309, cell signalling |
| Phospho-RB | Rabbit | 1:1000 | D59B7, cell signalling |
| CDK2 | Rabbit | 1:1000 | 2546,cell signalling |
| E-cadherin | Rabbit | 1:1000 | 3195, cell signalling |
| C-MYC | Rabbit | 1:1000 | 5605, cell signalling |
| Phospho-C-MYC | Rabbit | 1:1000 | 13748,cell signalling |
| Cyclin E | Mouse | 1:1000 | Sc-248,santa cruz |
| Caspase-3 | Rabbit | 1:1000 | 9662, cell signaling |
| Beta actin | Rabbit | 1:5000 | LF-PA0207,AbFrontier |
| GAPDH | Rabbit | 1:5000 | LF-PA0212,AbFrontier |
| Anti-Rabbit HRP | Goat | 1:5000 | AP132P,Millipore |
| Anti-Mouse HRP | Goat | 1:5000 | GTX213111-01, GeneTex |

Supplementary Table S2. Primers used for qRT-PCR

| **Primer name** | **Orientation** | **Sequence** |
|---|---|---|
| E-Cadherin | Sense | 5′-GGT CGA CAA AGG ACA GCC TA-3′ |
| | Anti-sense | 5′-GCG TGA CTT TGG TGG AAA AC-3′ |
| N-Cahderin | Sense | 5′-CAA CCC CAT CTC GGG TCA GC-3′ |
| | Anti-sense | 5′-GGG CAT TGG GAT CGT CAG CA-3′ |
| ZEB1 | Sense | 5′-CAG CAT CAC CAG GCA GTC CC-3′ |
| | Anti-sense | 5′-CAA CAG CTT GCA CCA TGC CC-3′ |
| Vimentin | Sense | 5′-CCC TCA CCT GTG AAG TGG AT-3′ |
| | Anti-sense | 5′-TCC AGC AGC TTC CTG TAG GT-3′ |
| CDK2 | Sense | 5′-AGA CTG AGG GTG TGC CCA GT-3′ |
| | Anti-sense | 5′-AAG GTC TCG GTG GAG GAC CC-3′ |
| MYC | Sense | 5′-AGA GAA GCT GGC CTC CTA CC-3′ |
| | Anti-sense | 5′-CGT CGA GGA GAG CAG AGA AT-3′ |
| TERT | Sense | 5′-TGT CAA GGT GGA TGT GAC G-3′ |
| | Anti-sense | 5′-CTG GAG GTC TGT CAA GGT A-3′ |
| Beta -actin | Sense | 5′-AGA GCT ACG AGC TGC CTG AC-3′ |
| | Anti-sense | 5′-AGC ACT GTG TTG GCG TAC AG-3′ |

Supplementary Table S3. A list of 38 breast cancer cell lines in CCLE data base

| |
|---|
| BT-20 ,HCC70, BT-474, Hs 578T, BT-549, KPL-1, CAL-51,MCF7, CAMA-1, MDA-MB-134-VI, DU4475, MDA-MB-157, EFM-192A, MDA-MB-175-VII, EFM-19, MDA-MB-231, HCC1143, MDA-MB-361, HCC1187, MDA-MB-415, HCC1395, MDA-MB-436, HCC1419, MDA-MB-453, HCC1500, MDA-MB-468, HCC1569, SK-BR-3, HCC1806, T47D, HCC1937, UACC-812, HCC1954, UACC-893, HCC202, ZR-75-1, HCC2218, ZR-75-30 |

Supplementary Table S4. Patient characteristics in four public mRNA expression data sets

| **Data set (patients no.)** | **GSE6532 (N=261)** | **GSE26971 (N=210)** | **GSE2034 (N=209)** | **GSE113863 (N=181)** |
|---|---|---|---|---|
| HR | | | | |
| Positive | 261 | 210 | 209 | 181 |
| Negative | 0 | 0 | 0 | 0 |
| pTstage | | | | |
| T1 | 122 | 82 | NA | NA |
| T2 | 139 | 122 | NA | NA |
| T3 | 0 | 6 | | |
| pNstage | | | | |
| N0 | NA | 126 | NA | NA |
| N1 | NA | 46 | NA | NA |
| N2 | NA | 18 | NA | NA |
| N3 | NA | 20 | NA | NA |
| LN metastasis | | | | |
| Yes | 79 | 126 | 209 | NA |
| No | 182 | 84 | 0 | NA |
| Distant recurrence | | | | |
| Yes | 59 | 44 | 80 | 14 |
| No | 202 | 166 | 129 | 167 |
| Median F/U (yr) | 5.91 | 6.21 | 7.17 | 5.58 |

HR, Hormone Receptor; LN, Lymph Node; NA, Non-Available; F/U, Follow Up; yr, year; Staging is based on American Joint Committee on Cancer staging revised in 2002.

Supplementary Table S5. Multivariate analysis of prognostic factors for distant recurrence-free survival in hormone receptor-positive breast cancer

| **Data set** | **Variables** | | **DRFS** | |
|---|---|---|---|---|
| | | | HR (95% CI) | *p* |
| GSE6532 | | | | |
| | CCNE1 | (low vs high) | 1.93(1.13-3.32) | **0.017** |
| | pTstage | (T1 vs T2) | 2.90(1.61-5.21) | **<0.001** |
| | LN metastasis | (no vs yes) | 1.75(1.03-2.99) | **0.039** |
| GSE26971 | | | | |
| | CCNE1 | (low vs high) | 2.48(1.33-4.62) | **0.004** |
| | pTstage | (T1 vs T2) | 1.52(0.74-3.12) | 0.249 |
| | | (T1 vs T3) | 3.53(0.92-13.60) | 0.066 |
| | pNstage | (N0 vs N1) | 0.69(0.28-1.69) | 0.686 |
| | | (N0 vs N1) | 1.28(0.47-3.44) | 0.632 |
| | | (N0 vs N1) | 3.11(1.31-7.38) | **0.010** |

DRFS, Distant Recurrence-Free Survival; HR, hazard ratio; CI, confidence interval; Criteria of CCNE 'low vs high' are 'median' in the GSE6532 and '75 percentile' in the GSE26971; Staging is based on American Joint Committee on Cancer staging revised in 2002.

© 2020 by the authors. Licensee MDPI, Basel, Switzerland. This article is an open access article distributed under the terms and conditions of the Creative Commons Attribution (CC BY) license (http://creativecommons.org/licenses/by/4.0/).
